# Population genomic variation in Staphylococcus aureus isolates from carriage and disease in Indigenous communities in the Southwest United States

**DOI:** 10.1099/mgen.0.001666

**Published:** 2026-03-23

**Authors:** Eleonora Cella, Catherine G. Sutcliffe, Del Yazzie, Annaliesa S. Anderson, Lubomira Andrew, Ladonna Becenti, George Brasinikas, Loretta Christensen, Wanda Cowboy, Sheri L. Dixon, Lindsay R. Grant, Li Hao, Robin Hubler, Hal Jones, Pierrette Montanez, Dennie Parker, Urvi Rajyaguru, Amy Rice, Eugene Romancito, Charis Salabye, Valerie L. Tenequer, Polly Thompson, Minnie Tsingine, Carol Tso, Dan VanDeRiet, Laura L. Hammitt, Taj Azarian

**Affiliations:** 1 Univ. of Central Florida, Orlando, FL, USA; 2 Johns Hopkins Bloomberg School of Public Health, Baltimore, MD, USA; 3 Navajo Epidemiology Center, Window Rock, AZ 86515, USA; 4 Pfizer Vaccines Research & Development, Pearl River, NY, USA; 5 Gallup Indian Medical Center, Indian Health Service, Gallup, NM, USA; 6 Indian Health Service, Rockville, MD, USA; 7 Tséhootsooí Medical Center, Fort Defiance, Arizona, USA; 8 Pfizer Vaccine Medical Development, Scientific and Clinical Affairs, Pfizer Inc, Collegeville, PA, USA; 9 Northern Navajo Medical Center, Indian Health Service, Shiprock, NM, USA; 10 Crownpoint Health Care Facility, Indian Health Service, Crownpoint, NM, USA; 11 Whiteriver Indian Hospital, Indian Health Service, Whiteriver, AZ, USA; 12 Tuba City Regional Health Care Corporation, Tuba City, AZ, USA; 13 Kayenta Health Center, Kayenta, AZ, USA; 14 Chinle Comprehensive Health Care Facility, Indian Health Service, Chinle, AZ, USA; 15 Winslow Indian Health Care Center, Winslow, AZ, USA

**Keywords:** carriage, Indigenous health, invasive disease, phylogenomic analysis, *Staphylococcus aureus*

## Abstract

Indigenous populations in the USA experience disproportionately high rates of *Staphylococcus aureus* invasive disease, yet the drivers of this disparity remain unclear. To investigate the role of bacterial population structure, we conducted whole-genome sequencing of 589 invasive *S. aureus* isolates and 125 carriage isolates collected from the Navajo Nation and White Mountain Apache (N/WMA) tribal lands in the Southwest USA. We compared lineage distribution between invasive disease and carriage, and to a contemporaneous set of bloodstream isolates from New Hampshire (NH) (*n*=377), using a Monte Carlo simulation based on the relative frequency ratio (RFR). Methicillin-resistant *S. aureus* accounted for 33.4% of invasive infections in N/WMA, and the most prevalent lineages were multi-locus sequence types (STs) 8 and 5. ST8 and ST97 were significantly enriched in invasive disease relative to carriage in N/WMA, suggesting increased invasiveness. Compared to NH, invasive disease in N/WMA was associated with higher frequencies of ST8, ST97 and ST188, while ST5 and ST30 were more common in NH. These findings highlight substantial geographic variation in *S. aureus* population structure and suggest that lineage composition may contribute to the elevated burden of invasive disease in Indigenous communities in the Southwest US. Our study underscores the importance of integrating genomic surveillance with epidemiologic data to inform prevention strategies.

**Impact Statement**

*Staphylococcus aureus* is a human commensal and pathogen that causes a wide range of infections. Indigenous populations in the USA experience disproportionately high rates of *S. aureus* invasive disease, yet the drivers of this disparity remain unclear. To investigate the role of bacterial population structure, we sequenced 589 invasive *S. aureus* isolates and 125 carriage isolates collected from the Navajo Nation and White Mountain Apache tribal lands in the Southwest USA. We compared lineage distribution between invasive disease and carriage and to a contemporaneous set of 377 bloodstream isolates from New Hampshire. We found substantial variation in *S. aureus* population structure between invasive disease and carriage and between geographic regions, suggesting that lineage composition may contribute to the elevated burden of invasive disease in Indigenous communities in the Southwest US. Our study underscores the importance of integrating genomic surveillance with epidemiologic data to inform prevention strategies.

## Data Summary

The *Staphylococcus aureus* genome sequences are available from the National Center for Biotechnology Information Sequence Read Archive (BioProject number: PRJNA1280675). The accession numbers are included in File S1, available in the online Supplementary Material. Participant data collected from Indigenous individuals are governed by the participating Tribal Nations. Data can be made available upon request (contact lhammitt@jhu.edu or csutcli1@jhu.edu), if consistent with the Institutional Review Board-approved protocol and if the disclosure is approved by the participating Tribes.

## Introduction

*Staphylococcus aureus* is a human commensal and pathogen that causes a wide range of infections from mild skin and soft tissue infections to severe invasive disease, including bacteraemia and pneumonia [1,3]. Methicillin-resistant *S. aureus* (MRSA) infections are particularly difficult to treat, leading to increased morbidity, mortality and healthcare costs [4]. In 2017, an estimated 119,247 cases of *S. aureus* bloodstream infections and 19,832 associated deaths occurred in the USA, the most recent year with available data [5]. While rates of MRSA bloodstream infections have declined, community-onset infections from methicillin-susceptible *S. aureus* (MSSA) are increasing [6].

Globally, *S. aureus* invasive disease and carriage rates among Indigenous populations, including in the USA, are higher than in the general population [7,10]. Recent data from 2016 to 2018 found that rates of invasive MRSA infections among Indigenous individuals living in tribal lands in the Southwest US were up to seven times the national average [9,10]. As asymptomatic carriage of *S. aureus* on the skin or in the nasal passage is a significant risk factor for developing disease [11,12], we previously conducted a study of *S. aureus* carriage in these same communities in the Southwest. We estimated *S. aureus*/MRSA carriage prevalence in 2017 at 20.7%/1.7% among children, 30.2%/2.8% among adults aged 18–64 years and 16.7%/3.3% among adults ≥65 years [13], which is not dissimilar to estimates from the general USA population. The disparity in invasive disease rates is therefore not explained by differences in carriage; however, *S. aureus* population composition may be a contributing factor.

The population structure of *S. aureus* is commonly defined using multi-locus sequence types (STs), which are grouped into broader clonal complexes (CCs) [14]. Phylogenetic analysis supports this classification, with STs generally corresponding to major clades, notwithstanding some exceptions due to polyphyly. Henceforth referred to as lineages, STs differ considerably in genome content [15], including the presence of mobile genetic elements such as bacteriophages, integrative and conjugative elements, plasmids and polymorphic antigens, which encode factors important for niche adaptation and transmission dynamics [16]. Such genomic differences contribute to variation in invasiveness, antibiotic resistance and lineage success [17]. The distribution of * S. aureus* lineages also varies geographically – for example, ST8 predominates in North America, while ST22 (EMRSA-15) and ST30 (EMRSA-16) are more common in the UK [18]. While MRSA has historically been prioritized in surveillance efforts due to its clinical importance, MSSA is increasingly recognized as a major cause of invasive disease [5]. However, the population structure and relative invasiveness of MSSA lineages remain understudied, particularly in community settings and underrepresented populations.

Here, we investigate the population genomics and longitudinal trends in *S. aureus* invasive disease in Indigenous communities in the Southwest USA. Through population genomic analysis for whole-genome sequencing (WGS) data, we compared invasive isolates to carriage isolates collected from the same communities [13] to assess lineage-specific contributions to disease and identify associated virulence and resistance determinants. We also compared bacteraemia isolates from these communities to a contemporaneous dataset from a separate US study [19] to explore geographic variation in the population structure of disease-causing lineages.

## Methods

### Ethics statement

The study was reviewed and approved by participating Tribal communities, the Johns Hopkins Bloomberg School of Public Health Institutional Review Board (IRB; #6771), the Indian Health Service Phoenix Area IRB (PXR 16.02), the Indian Health Service National IRB (N16-N-02) and the Navajo Nation Human Research Review Board (NNR-16.238). An IRB-approved Health Insurance Portability and Accountability Act waiver was obtained to conduct the chart reviews.

### Surveillance for invasive *S. aureus* disease

This analysis was conducted within the Johns Hopkins Center for Indigenous Health (JHU-CIH) Active Bacterial Surveillance system, which has been previously described [9,10]. Briefly, active laboratory and population-based surveillance for *S. aureus* invasive disease was initiated in the Navajo Nation and White Mountain Apache (N/WMA) Tribal lands in May 2016. Cases were identified by contacting clinical microbiology laboratories at Indian Health Service, Tribal Health and private healthcare facilities in or near the N/WMA Tribal lands. When a case was identified, *S. aureus* isolates were sent to the JHU-CIH laboratory in Whiteriver, AZ, where they were confirmed using BBL CHROMagar *Staph aureus* plates (Franklin Lakes, NJ) and stored at −80 °C. For each case, a chart review was conducted to collect demographics, underlying medical conditions, clinical syndrome, antimicrobial resistance (AMR) test results and health outcomes. A case of invasive *S. aureus* infection was defined as an individual living in or near the N/WMA Tribal lands with *S. aureus* isolated from a normally sterile body site (e.g. blood, cerebrospinal fluid, pleural fluid, synovial fluid and deep tissue). Individuals may have contributed isolates from different body sources on the same day (counted as a single case) or isolates collected on different days (isolates collected <29 days after the initial culture were considered part of the same case). Infections were classified as hospital-onset (HO) if specimens were collected >3 days after admission (with admission as day 1). Healthcare-associated community-onset (HACO) was defined as cases with a healthcare risk factor (e.g. dialysis, surgery, hospitalization, long-term care within the past 12 months or vascular catheter use within 2 days), with specimens collected ≤3 days after admission. All remaining cases were considered community-associated (CA).

### Whole-genome sequencing

WGS of invasive isolates was performed by Pfizer Inc. as previously described [20]. Genomic DNA was extracted from cell suspensions using magnetic bead technology (Agencourt Genfind V2, Beckman Coulter, Brea, CA), resulting in a final elution in 100 µl of 10 mM Tris, 1 mM EDTA, pH 8 buffer. DNA concentration was then measured using the Qubit dsDNA HS Assay Kit (Life Technologies, Carlsbad, CA) and adjusted to a final concentration of 0.2 ng µl^−1^ with dH_2_O. The Nextera XT DNA Sample preparation protocol (Illumina, San Diego, CA) was followed to prepare DNA libraries for WGS. Nextera XT v2, 2×300 bp paired-end library quality was evaluated using the Agilent High Sensitivity DNA Kit (product # G2938-90321, Agilent Technologies, Santa Clara, CA). The library was then normalized per the manufacturer’s instruction (Illumina), evaluated using Power SYBR Green qPCR (Life Technologies, Norwalk, CT) and sequenced on an Illumina MiSeq platform. *De novo* assembly of short-read WGS data was performed using Unicycler v0.4.8 with default options [21], and pangenome analysis was performed using Roary v.3.12 using default parameters [22]. A maximum-likelihood phylogeny was inferred from a concatenated alignment of core orthologous genes (core genes) with iq-tree v2 [23] using the ASC_GTRGAMMA substitution model with 1,000 bootstrap replicates, and population structure was assessed using fastBAPS [24]. AMRFinderPlus was used to identify acquired antibiotic resistance and virulence genes, as well as genetic mutations known to confer AMR [25]. ABRicate v.1.0.1 was used to identify plasmids in total or partial sequences (https://github.com/tseemann/abricate) [26] using the PlasmidFinder database [27]. Finally, for MRSA isolates, SCC*mec* type was determined by assessing *ccr* and *mec* gene complexes from assemblies using the SCC*mec* finder tool (www.genomicepidemiology.org/). Isolates with a *mecA* gene and SCC*mec* typing were genotypically defined as MRSA. Differences in acquired AMR determinants and virulence genes were tested using chi-square or Fisher’s exact test when applicable. All data analytics were performed using R scripts and results visualized using the *ggplot* libraries in R v.3.6 [28,29].

### Comparison datasets

To determine the relative contribution of lineages to disease, we compared the N/WMA invasive isolates to carriage isolates collected in 2017 from children <5 years (*n*=24) and adults ≥18 years (*n*=101) in the same communities in the N/WMA Tribal lands [13]. For this analysis, invasive isolates were limited to the same time period as the carriage study, resulting in 125 and 195 isolates from carriage and invasive disease, respectively. The 125 carriage isolates were identified from 91 participants [70.3% (*n*=64) were female, and 74.7% (*n*=68) were adults]. In instances where genotypically distinct carriage isolates were identified from multiple anatomical sites of the same participant, one representative isolate from each lineage was included in the analysis [30].

To investigate spatial variation in population structure of *S. aureus* lineages causing invasive disease in another USA region, we compared the N/WMA invasive isolates described above [9,10] with a recently published genomic epidemiology study of invasive disease in New Hampshire (NH) [19]. The NH dataset consisted of *S. aureus* blood isolates collected from paediatric and adult patients diagnosed with bloodstream infections from 2010 to 2018 [19]. To reduce temporal bias, we limited the analysis to blood isolates collected during 2016–2018 from each dataset, resulting in 189 NH isolates and 376 N/WMA isolates.

To compare *S. aureus* population structures between groups, we used nonparametric bootstrap resampling to estimate lineage-level differences in relative frequency. For each comparison (invasive N/WMA vs invasive NH and invasive vs carriage within N/WMA), we repeatedly resampled isolates within each group with replacement to generate pseudo samples of fixed size (*n*=100). In each resample, we calculated the proportion of isolates assigned to each CC or ST. For each lineage, we computed the relative frequency ratio (RFR) between groups as the ratio of these proportions and summarized the bootstrap distribution to obtain a point estimate (median RFR) and a bootstrap percentile interval (BPI; 2.5th to 97.5th percentiles). We considered a lineage to differ between groups when the 95% bootstrap percentile interval for RFR excluded 1.0 (no difference). Lineages with very low total counts across the two comparison groups were excluded from inferential testing due to instability. The results were plotted to visualize significance for each variable of interest (https://github.com/sayfaldeen/BioinformaticsScripts).

As a sensitivity analysis to confirm that key findings were not driven by small cell counts, we used the raw data to perform Fisher’s exact tests comparing the proportion in a given lineage (e.g. a specific CC or ST present vs. not present) between groups. A two-sided *P*-value<0.05 was considered evidence of an association between group and lineage membership. We report Fisher’s exact *P*-values alongside RFR estimates in the Supplementary Material. When reporting results, we prioritized bootstrap RFRs as our primary effect size estimate and used Fisher’s exact tests as a sparse-data sensitivity analysis, emphasizing lineages supported by both approaches.

Following the initial population genomic analysis, we conducted a comprehensive investigation of the population structure of each dominant CC by examining phylogenies derived from CC-specific core gene alignments. To assess the population structure based on the study population, we employed a compartmentalization analysis using two tree-based statistical methods: correlation coefficients (r, rb) and the Simmonds association index (AI). These analyses were carried out using the HYPHY software [31]. We calculated correlation coefficients using either the number of branches (rb) or branch lengths (r) separating isolates from different populations [32]. Maximum-likelihood phylogenies were constructed using core gene alignments, employing the GTR nucleotide substitution model with gamma-distributed rate variation across sites (GTR+G). Observed values were compared to a null distribution generated by 1,000 sequence permutations. A correlation value >0.5 was considered evidence of significant compartmentalization. Simmonds AI was calculated based on the association value (d), defined as (1 − f)/(2n − 1), where n is the number of sequences below the node and f is the frequency of the most common sample type. Bootstrapping with 1,000 replicates was used to assess significant population compartmentalization [33].

## Results

### Invasive disease dataset description

Over a 3-year period (1 May 2016 to 30 April 2019), 719 isolates were identified from 659 cases of invasive disease in the N/WMA Tribal lands. The first isolate collected from an episode of invasive disease was selected for sequencing, resulting in 589 isolates. The median age of cases was 55 years (interquartile range, IQR: 43–66 years), 399 (67.7%) were male, 369 (63.2%) were diabetic, 187 (31.7%) had a history of previous *S. aureus* infection, 347 (58.9%) had an infection classified as HACO, and 39 (6.6%) of cases died from their infection (Table S1, available in the online Supplementary Material).

Of the 589 isolates, 197 (33.4%) were genotypically identified as MRSA. Among the 197 MRSA-classified isolates, 6 were phenotypically susceptible to oxacillin, and 11 were oxacillin-resistant but lacked *mecA*, thus reclassified as MSSA. The proportion of isolates that were MRSA varied by type of infection, with MRSA accounting for 42.4%, 38.3% and 23.9% of HO, HACO and CA infections, respectively (χ², *P*=0.001). A decrease in the proportion of MRSA among isolates from all sources was observed over time, from 44.4% in 2016 to 30.8% in 2019 (*P*=0.04) (Fig. S1A). No significant differences in the proportion of isolates identified as MRSA were found by age group (*P*=0.11).

**Fig. 1.**
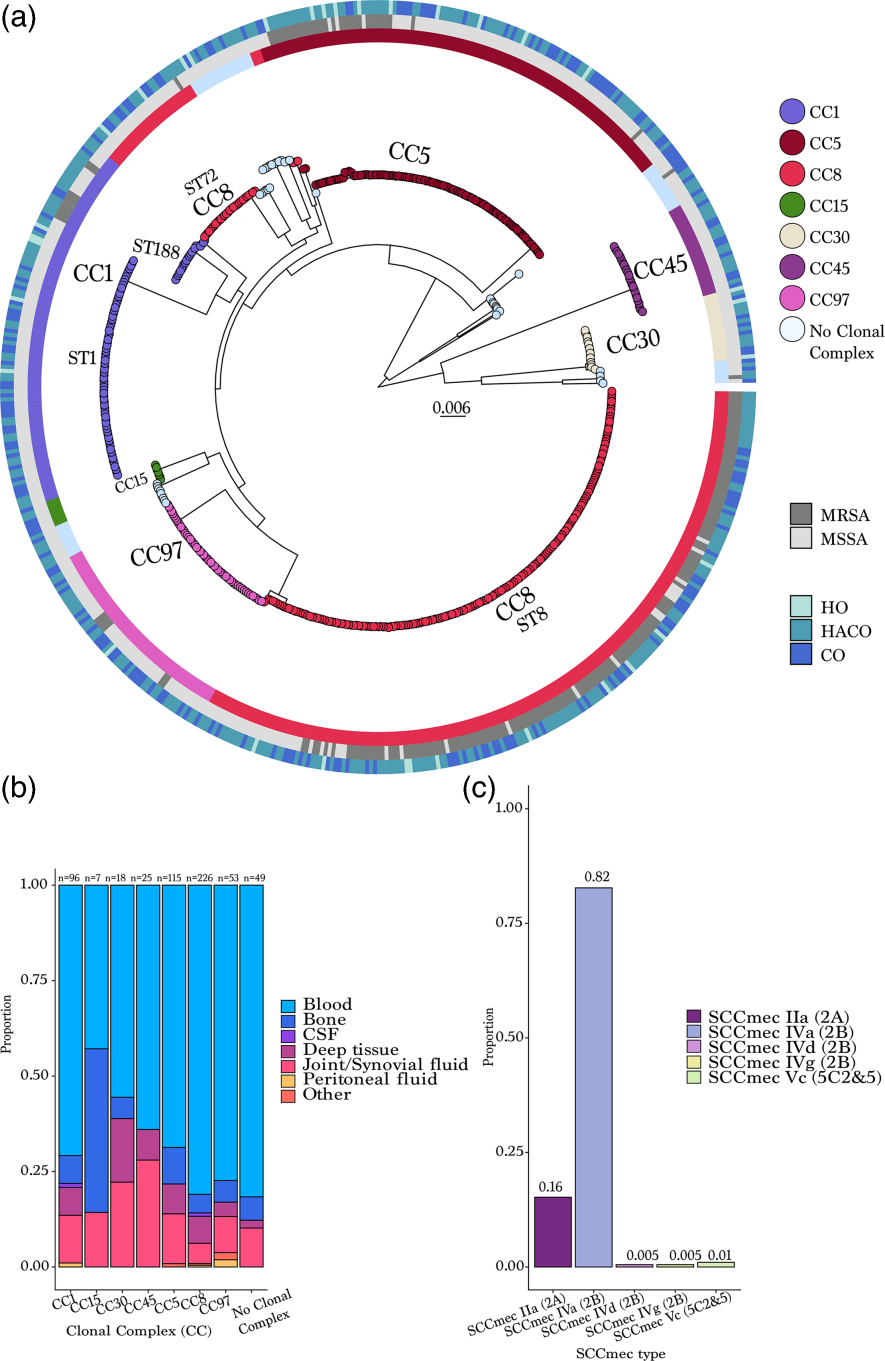
(a) Maximum likelihood phylogeny of 589 invasive disease isolates inferred from a concatenated alignment of core gene SNPs. Each tip corresponds to an isolate and is coloured according to the CC. Isolates labelled ‘No Clonal Complex’ are STs that are not assigned to a CC under the PubMLST (*S. aureus*) CC assignment. The first ring surrounding the tree denotes methicillin susceptibility and resistance (MSSA, MRSA). The external ring around the tree denotes whether the infection was classified as hospital-onset (HO), healthcare-associated community-onset (HACO) or CA. The legend indicates the CC, which is also labelled on the phylogeny. (**b**) Distribution of CCs and source of collection. Each bar is stratified by anatomical site of collection. (**c**) SCCmec types among 197 MRSA isolates.

Population genomic analysis of WGS data was conducted using the 589 invasive disease isolates. The maximum likelihood phylogeny inferred from a concatenated alignment of 1,626 core clusters of orthologous groups (CDSs shared among all isolates in the sample) and 62,035 SNPs resolved several well-supported clades representing the dominant CCs (Fig. 1a). The two most common CCs were CC8 (*n*=226, 38.4%) and CC5 (*n*=115, 19.5%) (Fig. 1a, b). CC8 primarily consisted of ST8 (77.9%) and ST72 (11.1%), while CC5 was largely made up of ST5 (78.3%). The population structure appeared relatively stable during the study years with stochastic fluctuations in the proportion of lineages (Fig. S1B). However, a slight increase in the proportion of CC8 MSSA was observed, partially explaining the overall decrease in the proportion of MRSA found in invasive disease from 2016 to 2019.

To explore lineage-specific tropism for clinical sources of invasive isolates, we assessed the association between CC and body sites. Compared to all other CCs, CC8 was significantly more likely to be isolated from blood (RFR=1.4, 95% BPI: 1.1–1.8), while CC30 (RFR=3.5, 95% BPI: 1.8–9.0) and CC45 (RFR=2.4, 95% BPI: 1.1–7.0) were more likely to be isolated from joint/synovial fluid (*P*<0.05 by resampling). Notably, the CC30 clade was bifurcated into two lineages, the commonly observed ST30 and a novel sequence type, ST7317. When stratified by STs, only ST8 (RFR=1.5, 95% BPI: 1.2–1.8) belonging to CC8 was more likely to be isolated from blood, while ST30 (RFR=3.3, 95% BPI: 1.6–10.5) belonging to CC30 and ST45 (RFR=2.6, 95% BPI: 1.3–5.7) belonging to CC45 were more likely to be isolated from joint/synovial fluid. Fisher’s exact tests performed as a sensitivity analysis were broadly consistent with the bootstrap RFR inference for the strongest signals, with some differences for moderate-effect or sparse lineages. MRSA and MSSA isolates showed no significant differences in site of infection. Among MRSA strains (*n*=197), the most common SCC*mec* type found in 163 isolates (82.7%) was IVa (2B), followed by IIa (2A) (*n*=30, 15.2%), Vc (5C and 5) (*n*=2, 1.0%), IVd (2B) (*n*=1, 0.5%) and IVg (2B) (*n*=1, 0.5%) (Fig. 1c). These associations should be interpreted in the context of the active, population-based surveillance design used to capture invasive cases across multiple clinical laboratories serving the catchment area. Although the surveillance approach is intended to minimize ascertainment differences across facilities, we cannot fully exclude residual confounding from unmeasured facility-level effects or site-specific clinical sampling practices.

We observed broadly similar CC distributions between isolates collected from the Navajo Nation and WMA Tribal lands, but several lineage differences were evident (see Table S2). The CC8/ST8 lineage accounted for a higher proportion of isolates in the WMA Tribal lands and counted for a higher proportion of MRSA, whereas ST188 (CC1) was enriched in the Navajo Nation (Table S2). CC97 (ST97) and CC5 (ST5) also trended higher in the Navajo Nation. Differences for CC1 and CC5 were supported by bootstrap RFR estimates but were not consistently significant by Fisher’s exact test, and we therefore interpret those as suggestive.

### Comparison of invasive disease with carriage isolates from the N/WMA Tribal lands

**Fig. 2.**
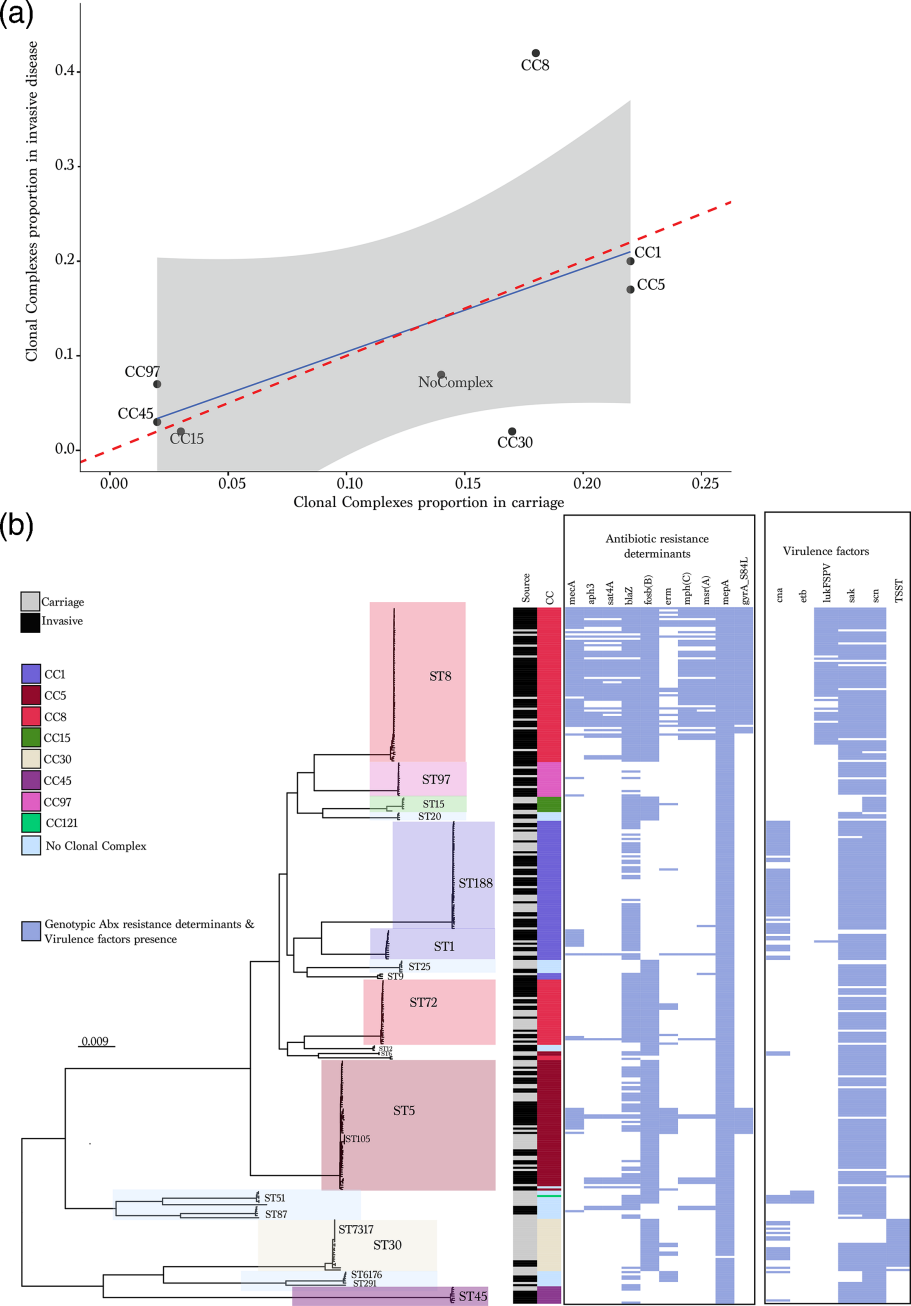
(a) The correlation between the proportion of CCs in invasive disease and carriage. (**b**) Maximum-likelihood phylogeny inferred from a concatenated core gene SNP alignment of invasive disease (*n*=195) and carriage (*n*=125) isolates. Clades are highlighted by CC. The legend corresponds to the collection source (carriage/invasive disease), CC and the presence/absence of acquired antibiotic (abx) resistance genes and virulence factors, which are shown in the heatmap. Antibiotic resistance genes in order: *mecA*, *aph3.III*, *sat4A*, *blaZ*, *fosb*(B), *erm*, *mphC*, *msrA*, *mepA* and *gyrA*. Virulence factors in order: chp, cna, etb, LukFS-PV, sak, scn and TSST. No Clonal Complex included all the STs that do not belong to any CC and novel STs.

While all lineages observed among N/WMA invasive isolates (*n*=195) were also detected in N/WMA carriage isolates (*n*=125) in 2017, comparative analysis showed clear enrichment of CC8/ST8 among invasive disease isolates (Fig. 2a and Table S3). CC30 was less frequent among invasive isolates than carriage. Several additional lineages (including ST97, ST5 and ST188) showed directional differences in relative frequency between invasive disease and carriage by bootstrap RFRs, but these were not uniformly supported by Fisher’s exact test and are therefore interpreted as suggestive.

Phylogenetic analysis of the 320 isolates revealed distinct lineage clustering between invasive disease and carriage, with acquired antibiotic resistance and virulence genes largely confined to specific STs (Fig. 2b). MRSA was significantly more prevalent among invasive isolates (34.8%) than carriage isolates (7.2%, *P*<0.0001). The *mecA* gene was primarily associated with ST1, ST5 and ST8. ST8 strains, in particular, exhibited a high burden of resistance genes. Notably, acquired macrolide resistance determinants were among the most prominent in the dataset, including *erm*, *mphC* and *msrA* (Fig. 2b), indicating that macrolide resistance is not confined to MRSA backgrounds in these populations. Moreover, the majority of ST8 and a subset of ST5 strains carried the Ser84→Leu substitution in *gyrA* that confers fluoroquinolone resistance. ST8 strains also harboured the *lukSF-PV* locus encoding Panton–Valentine leucocidin, which was exclusive to this lineage. In contrast, most other lineages, especially those enriched in carriage, lacked major resistance elements. The toxic shock syndrome toxin gene (TSST-1) was found almost exclusively in CC30 MSSA strains from both carriage and disease, though only a minority of these were associated with invasive cases (*n*=3). Overall, 79.4% (*n*=254) of isolates carried at least 1 replicon gene, with 40.3% harbouring 2–3 distinct replicons. The most common types were plasmids rep7c (37.8%) and rep16 (31.6%). The rep7c replicon was identified largely in CC1 (*n*=14, 20.9%) and CC8 (*n*=101, 98.1%), while rep16_2 (pSJH101) replicon was found in >54% of CC8 (56 out of 103), CC30 (20 out of 24) and CC121 (1 out of 1). Further analysis of plasmid replicons and associated AMR determinants showed that *msr*(A), *mph*(C), *blaZ*, *mepA* and *fosB*(B) were associated with multiple replicon types, most frequently rep10, rep19, rep7c, rep16_2 and rep5.

### Comparison of invasive disease isolates across geographic settings

Invasive blood isolates collected from N/WMA and NH between 2016 and 2018 showed significant differences in CC distribution. ST8, ST97 and ST188 were more frequently identified in N/WMA cases, while ST5, ST30 and ST105 were more common among NH isolates (Table S4). These findings highlight geographic variation in lineage prevalence among *S. aureus* bloodstream infections.

**Fig. 3.**
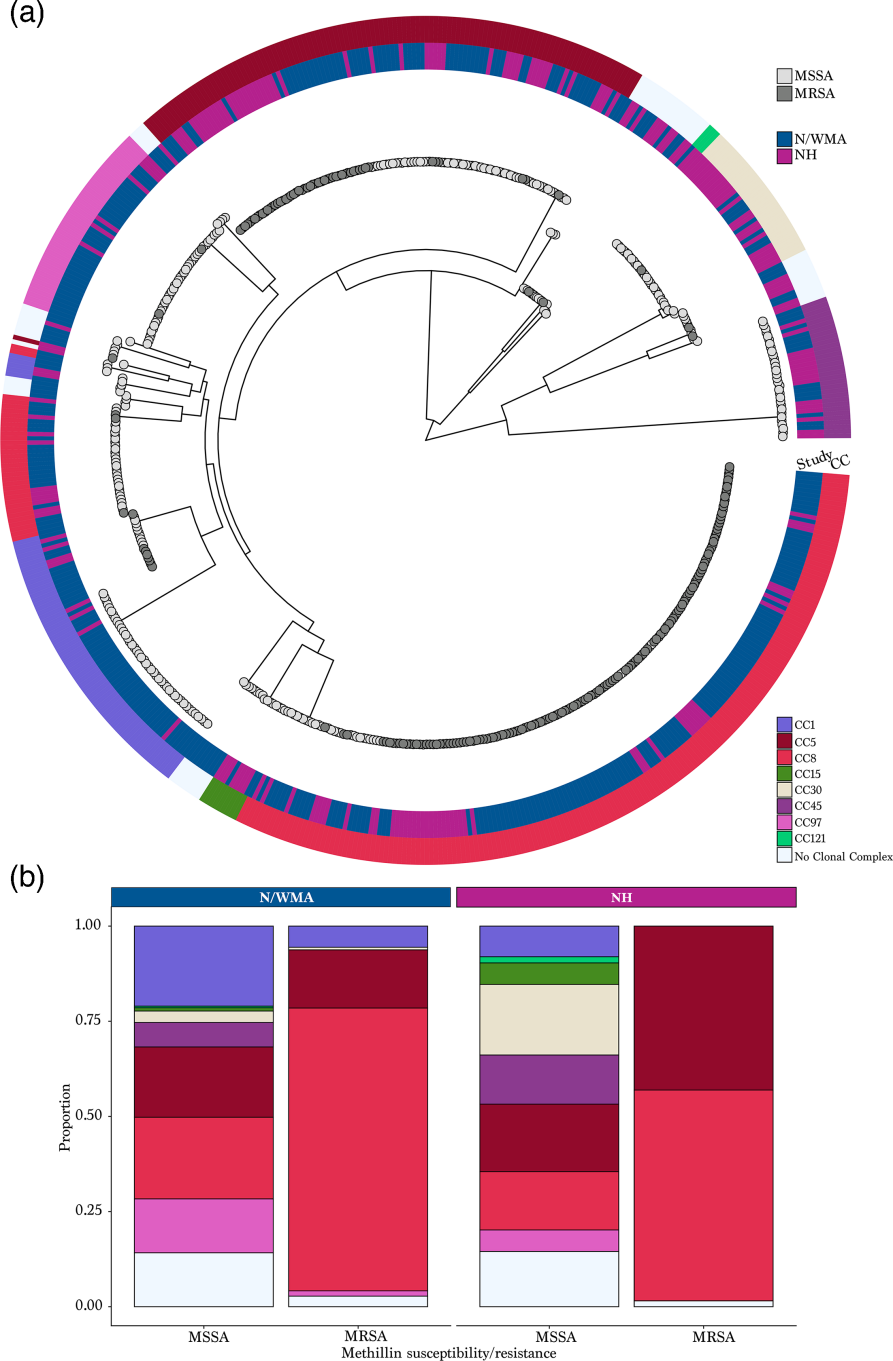
(a) Maximum-likelihood phylogeny inferred using a concatenated core gene SNP alignment of 583 *S. aureus* isolates (New Hampshire, NH cases, *n*=189, Navajo/White Mountain Apache, N/WMA cases, *n*=376). Each tip of the phylogeny is colour-coded based on the methicillin susceptibility/resistance. The first ring surrounding the tree denotes the study population. The external ring around the tree denotes CC of each isolate, with colour legend provided on the right side of the figure. (**b**) A comparison of the distribution of CC is shown stratified by methicillin susceptibility and study population. No Clonal Complex included all the STs that do not belong to any CC and novel STs.

Phylogenies revealed distinct clades corresponding to dominant CCs and identified population-specific clusters, consistent with geographic compartmentalization (Fig. 3a). The overall MRSA proportion was similar between N/WMA and NH (38.3% vs. 34.4%, *P*=0.38); however, MRSA composition differed. While CC8 dominated in both regions, CC5 MRSA was more prevalent in NH, whereas CC1 MRSA was exclusive to N/WMA (Fig. 3b). A comparison of MRSA-only isolates between the two population samples confirmed significant differences in CC (*P*=0.0005).

**Fig. 4.**
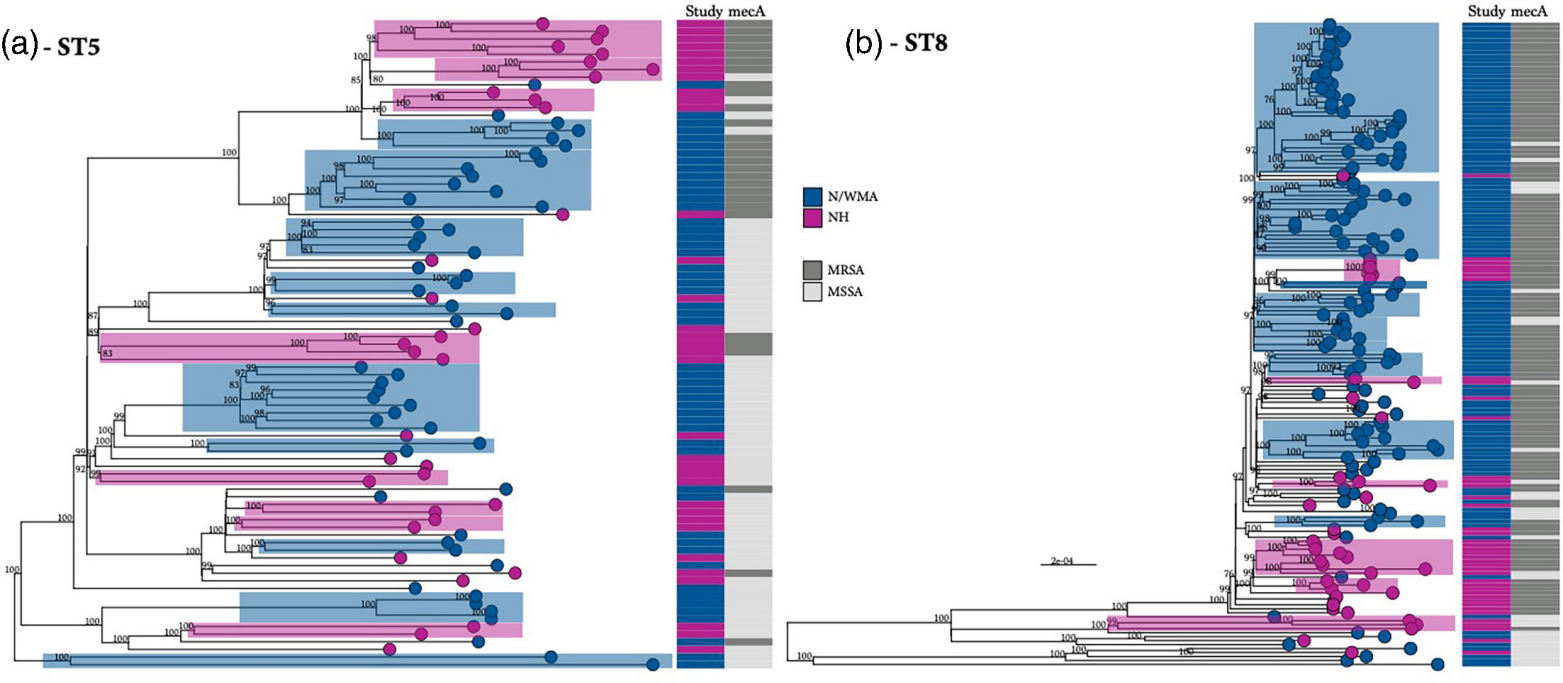
Comparison of population structure of dominant *S. aureus* lineages found in the Navajo/White Mountain Apache (N/WMA) and New Hampshire (NH) samples [ST5 – panel (a) and ST8 – panel (b)]. The tips of the phylogeny are coloured according to the study population. Bootstrap values for statistical support are annotated on the branches of the phylogeny, and the branch length scales are shown at the bottom of each panel. The heatmap to the right shows the study sample and methicillin-susceptible (MSSA) and methicillin-resistant (MRSA) *S. aureus*.

To assess lineage-specific population structure, we constructed separate phylogenies for ST5 and ST8 (Fig. 4). Both lineages showed clear geographic compartmentalization, with well-supported clades corresponding to either N/WMA or NH cases. In ST5, population-specific clusters were often restricted to either MRSA or MSSA. Core gene SNP distances further supported this structure: between-population diversity in ST5 (281.9 SNPs) exceeded within-population diversity (278.5 SNPs vs 277.1 SNPs for the NH and N/WMA populations, respectively). In ST8, within-population diversity was greater in NH (150.9 SNPs) than in N/WMA (122.1 SNPs), while between-population diversity (141.6 SNPs) fell between the two. This pattern was driven in part by MSSA outliers in the N/WMA sample.

## Discussion

This study provides a detailed comparative genomic analysis of *S. aureus* isolates collected from both invasive disease and asymptomatic carriage within Indigenous adults and children in the Southwest USA, enabling detailed examination of lineage-specific associations with clinical infection. Our integrated approach, linking WGS data with detailed clinical and epidemiologic metadata, revealed that a limited number of dominant lineages, particularly ST8 and ST5, account for the majority of invasive disease, with ST8 strains carrying a high burden of antibiotic resistance and virulence genes. These findings are consistent with the widespread circulation of USA300-like ST8 strains in the USA. ST8 was consistently enriched among invasive disease isolates relative to carriage across both bootstrap RFR and Fisher’s exact sensitivity analyses. Other lineages, including ST97, showed directional differences between disease and carriage in bootstrap analyses, but these were not uniformly supported by Fisher’s exact testing. These findings suggest that *S. aureus* population composition may be contributing to the high rates of invasive disease in these communities. In contrast, MSSA strains exhibited broader lineage diversity, and several lineages, most notably ST30, were more frequently recovered from carriage than disease, with additional lineages such as ST5 and ST188 showing similar directional patterns, suggesting reduced invasive potential for some MSSA backgrounds. Additionally, geographic comparisons revealed population structure and phylogenetic compartmentalization within both ST5 and ST8, with distinct region-specific clades and differences in core gene diversity between samples. These results offer a high-resolution view of *S. aureus* population structure across clinical and geographic contexts, supporting future genomic epidemiologic studies and informing future efforts in regional surveillance and lineage-specific risk assessment.

Only 3% of invasive *S. aureus* cases in our study were HO, with most infections classified as either CA or HACO. This pattern, along with more than 70% of cases presenting as bacteremia, suggests that *S. aureus* circulates extensively in the community and contributes significantly to bloodstream infections. The relatively low proportion of MRSA (33.4%) aligns with national data showing a rise in community-onset MSSA bacteraemia in the USA [5] and mirrors carriage data from these same communities, where MRSA was uncommon across all age groups [13]. We observed a high proportion of MRSA in invasive cases among individuals ≥65 years of age, consistent with increased healthcare contact and age-associated risk factors. The high burden of invasive disease in these populations may also reflect the impact of prevalent comorbidities, such as diabetes and obesity, and frequent healthcare interactions [9]. These factors are known to increase susceptibility to skin and soft tissue infections, which may serve as a source for subsequent bacteraemia.

The distribution of *S. aureus* lineages is known to vary geographically and over time. We hypothesized that elevated rates of invasive disease among Indigenous populations in the Southwest USA could be partially attributed to the prevalence of more virulent or antibiotic-resistant lineages. Among bacteraemia isolates, we observed significant geographic differences in lineage composition, particularly in the proportions of ST188 (CC1), ST8 (CC8), ST5 and ST105 (CC5), ST30 (CC30) and ST97 (CC97) between the N/WMA and NH samples [19]. ST8 and ST5, both previously associated with bloodstream infections [19,34], accounted for nearly half of all isolates across both sites. However, their distribution differed significantly by region; ST8 was more common in N/WMA cases, while ST5 predominated in NH. Within MRSA isolates, ST8 made up a larger proportion in N/WMA, whereas ST5 was more common in NH. Strikingly, nearly all ST5 isolates from N/WMA were MSSA (Fig. 3).

Additional lineages showed evidence of geographic and epidemiologic relevance. ST188 (CC1), historically associated with Indigenous communities in the USA [35,36], was enriched in N/WMA cases despite its broader rarity in North America. Although CC1 was largely supplanted by CC8 (USA300) nationally [37,38], lineages such as ST1-MRSA and ST188-MSSA appear to have persisted in these communities since early molecular surveys nearly 25 years ago. ST97 (CC97), a lineage previously linked to livestock and increasingly associated with human infections [39,42], was also disproportionately found in N/WMA disease isolates. Despite its prior association with oxacillin and tetracycline resistance, only 2 of 32 ST97 isolates from N/WMA were MRSA, suggesting it is circulating as an emerging invasive MSSA lineage. ST97 was also significantly more common in disease than carriage, further suggesting increased invasive potential. Ongoing monitoring of ST97’s prevalence and genomic evolution will be critical to assess its potential as a sustained contributor to MSSA disease burden.

Phylogenetic analysis of ST5 and ST8 revealed region-specific clades, consistent with population compartmentalization and limited strain migration. Of note, ST72, though classically assigned to CC8, was phylogenetically distinct from ST8. Prior work suggests that ST72 may have emerged through recombination between CC5 and CC8, sharing alleles from both lineages [43]. Given its divergence, we assessed ST72 independently and propose that it may warrant reclassification as a distinct CC. These geographic and lineage-specific differences raise critical questions about the invasive potential of specific lineages and the role of population structure in shaping disease burden.

While epidemic *S. aureus* lineages are often defined by their association with severe or difficult-to-treat infections, the relative invasiveness of specific lineages remains poorly understood. As shown here and in prior studies [44,45], *S. aureus* population structure can vary even across small geographic areas, highlighting the importance of comparing carriage and disease isolates from the same population to infer invasive potential. In our analysis, ST8 and ST97 were disproportionately associated with invasive disease, while other lineages were more often identified in carriage. A substantial proportion of invasive isolates were MSSA, reinforcing the important and underrecognized role of MSSA in disease and reflecting a broader decline in MRSA prevalence in the USA [46,47] and globally [48]. However, certain MSSA strains, such as ST8, now mirror the resistance and virulence profiles typically associated with MRSA [49,51], suggesting a convergence in clinical impact despite differing resistance profiles. This was reflected by acquired AMR determinants observed in MSSA backgrounds, including those conferring macrolide resistance (*erm*, *mphC* and *msrA*) and, in a subset, additional acquired genes (Fig. 2b). Macrolide-associated genes are particularly notable given recent evidence that macrolide selection can shape *S. aureus* population structure through the spread of mobile resistance elements [52], consistent with the high proportion of replicons we observed in the sample. Although our findings suggest lineage-level differences in disease propensity, efforts to quantify invasiveness are limited by gaps in our understanding of strain-specific carriage duration [53,55]. Longitudinal studies incorporating bacterial genomics and host metadata are needed to resolve these dynamics and define how long particular lineages persist in colonized individuals. Importantly, all lineages responsible for invasive disease were also detected among asymptomatic carriers in the same communities, indicating that the strains capable of causing severe infection are already circulating in the population. This overlap underscores the public health implication that carriage of particular lineages, even if relatively uncommon, can serve as a reservoir for strains with increased invasive potential.

In summary, we evaluated the population structure of invasive *S. aureus* isolates collected from indigenous communities in the Southwest USA to investigate potential contributors to the elevated burden of invasive disease. By integrating genomic and clinical data, we characterized the relative contribution of specific lineages to disease and identified geographic variation in lineage composition among bloodstream infections. ST8 and ST97 emerged as lineages of particular concern due to their enrichment in invasive disease and, in the case of ST97, their potential emergence as an epidemic MSSA clone.

Our findings underscore the growing role of MSSA in invasive infections and highlight the need to expand genomic surveillance to include both MSSA and MRSA from carriage and disease sources. While MRSA has historically garnered more public health attention, our results emphasize the importance of broadening the focus. We also observed epidemiological differences among lineages and evidence of community transmission, particularly in the context of comorbid conditions and household dynamics, which may drive persistence and spread. This study provides a high-resolution genomic view of *S. aureus* population structure across epidemiological contexts and regions, offering a framework for future research on transmission, persistence and prevention.

## Supplementary material

10.1099/mgen.0.001666 Uncited Supplementary Material 1.

## Abbreviations

AI: association index
AMR: antimicrobial resistance
BPI: bootstrap percentile interval
CA: community-associated
CCs: clonal complexes
HACO: healthcare-associated community-onset
HO: hospital-onset
ICE: integrative and conjugative elements
JHU-CIH: Johns Hopkins Center for Indigenous Health
MRSA: methicillin-resistant *Staphylococcus aureus*
MSSA: methicillin-susceptible *Staphylococcus aureus*
NH: New Hampshire
N/WMA: Navajo Nation and White Mountain Apache
RFR: relative frequency ratio
SNP: single nucleotide polymorphism
STs: sequence types
WGS: whole-genome sequencing
