# Supplementary material for: Population genomic variation in Staphylococcus aureus isolates from carriage and disease in Indigenous communities in the Southwest United States
Source: Microb Genom. 2026 Mar 23;12(3):001666. doi: 10.1099/mgen.0.001666 (PMC13034078; doi:10.1099/mgen.0.001666)
Supplement: Uncited Supplementary Material 1. [file mgen-12-01666-s001.pdf]

Supplemental Material

Population genomic variation in *Staphylococcus aureus* isolates from carriage and disease in Indigenous communities in the Southwest United States

Supplemental Tables

Supplemental Table 1. Characteristics of cases of invasive *Staphylococcus aureus* disease in the Southwest United States, 2016-2019

Supplemental Table 2. Comparison of clonal complex (CC) and sequence type (ST) frequencies in isolates from invasive disease between Tribal Lands: Navajo Nation (n=516) and White Mountain Apache (n=73) Tribal lands (N/WMA).

Supplemental Table 3. Comparison of clonal complex (CC) and sequence type (ST) frequencies between isolates from carriage (n=125) and invasive disease (n=195) in the Navajo Nation and White Mountain Apache Tribal lands, 2017

Supplemental Table 4. Comparison of clonal complex (CC) and sequence type (ST) frequencies in isolates from cases of bacteremia between study populations: Navajo Nation and White Mountain Apache Tribal lands (N/WMA) (n=376) and New Hampshire (NH) (n=189), 2016-2018

Supplemental Figures

Supplemental Figure 1. Proportion of *Staphylococcus aureus* strains from invasive disease isolates from N/WMA Tribal lands that were methicillin resistant by A) collection year and B) collection year, clonal complex (CC) stratified by methicillin-susceptibility (MSSA) and resistance (MRSA).

Supplemental Files

Supplemental File 1. Accession numbers for carriage and invasive isolates

Supplemental Table 1. Characteristics of cases of invasive *Staphylococcus aureus* disease in the Southwest United States, 2016-2019

	All (n=589)		Age group (years) 0-17 (n=14)		18-64 (n=416)		≥65 (n=159)	
	N	%	N	%	N	%	N	%
Collection site[a]								
White Mountain Apache Tribal lands	73	12.4	4	28.6	60	14.4	9	5.7
Navajo Nation	516	87.6	10	71.4	356	85.6	150	94.3
Sex								
Male	399	67.7	10	71.4	290	69.7	99	62.3
Female	190	32.3	4	28.6	126	30.3	60	37.7
Underlying condition: MRSA risk factor[b]								
Abscess/boil[a]	66	11.3	3	21.4	52	12.6	11	6.9
Current smoker	38	6.5	0	.	37	9.0	1	0.6
Decubitus/pressure ulcer[a]	48	8.2	0	.	21	5.1	27	17.0
Diabetes[a]	369	63.2	1	7.1	256	62.3	112	70.4
Intravenous drug use[a]	11	1.9	0	.	11	2.7	0	.
Malignancy[a]	43	7.4	0	.	20	4.9	23	14.5
Obesity[a]	158	27.0	0	.	130	31.6	28	17.6
Clinical syndrome of the case[b]								
Sepsis[a]	190	32.3	2	14.3	126	30.3	62	39.0
Osteomyelitis	150	25.5	5	35.7	110	26.4	35	22.0
Cellulitis	115	19.5	4	28.6	85	20.4	26	16.4
Pneumonia	64	10.9	1	7.1	40	9.6	23	14.5
Arthritis	54	9.2	1	7.1	38	9.1	15	9.4
Endocarditis	26	4.4	1	7.1	16	3.8	9	5.7
Necrotizing fasciitis[b]	10	1.7	0	.	10	2.4	0	.
Meningitis	4	0.7	0	.	4	1.0	0	.
Peritonitis	4	0.7	0	.	3	0.7	1	0.6
Hospitalized	514	87.3	12	85.7	358	86.1	144	90.6
Died[a,c]	39	6.6	0	.	21	5.0	18	11.3
Previous documented *S. aureus* infection	187	31.7	3	21.4	136	32.7	48	30.2
Type of infection[a,d]								
Healthcare-associated community-onset	347	58.9	3	21.4	244	58.7	100	62.9
Community-associated	209	35.5	10	71.4	149	35.8	50	31.4
Hospital onset	33	5.6	1	7.1	23	5.5	9	5.7
Source of the isolate								
Blood	440	74.7	12	85.7	310	74.5	118	74.2
Joint/synovial fluid	61	10.4	2	14.3	43	10.3	16	10.1
Deep tissue	40	6.8	0	.	28	6.7	12	7.5
Bone	39	6.6	0	.	27	6.5	12	7.5
Cerebrospinal fluid	3	0.5	0	.	3	0.7	0	.
Other	3	0.5	0	.	2	0.5	1	0.6
Peritoneal fluid	3	0.5	0	.	3	0.7	0	.
Antimicrobial resistance of the isolate[e]								
Methicillin susceptible (MSSA)	392	66.6	7	50.0	287	69.0	98	61.6
Methicillin resistant (MRSA)	197	33.4	7	50.0	129	31.0	61	38.4

[a] $p<0.05$ for comparison across age groups using a chi-square test or Fisher's exact test

[b] Cases may have more than one underlying condition or clinical syndrome

[c] Necrotizing fasciitis missing for 1 case; vital status missing for 36 cases; previous *S. aureus* infection missing for 1 case

[d] Infections were defined as hospital-onset when isolates were obtained from specimens collected >3 days after admission (with admission being day 1); as healthcare-associated community-onset if cases had a healthcare risk factor (dialysis, surgery, hospitalization, or long-term care in the past year, or vascular catheter in the past 2 days) and isolates were obtained from specimens collected prior to or ≤3 days after hospital admission; or as community-associated for all other cases.

[e] Isolates with a mecA gene and SCCmec typing were genotypically defined as MRSA.

Supplemental Table 2. Comparison of clonal complex (CC) and sequence type (ST) frequencies in isolates from invasive disease between Tribal Lands: Navajo Nation (n=516) and White Mountain Apache (n=73) Tribal lands (N/WMA).

Clonal Complex	Collection site	Relative Frequency Ratio (95% BPI)	ST	Collection site	Relative Frequency Ratio (95% BPI)
CC1	*WMA (n=7)*	*Ref*	*ST1*	*WMA (n=4)*	*Ref*
				Navajo (n=13)	0.5 (0.2 -1.3)
	Navajo (n=89)	**1.9 (1.2 – 3.0)**	*ST188*	*WMA (n=2)*	*Ref*
				Navajo (n=68)	**5.7 (2.9 – 14.7)****
CC5	*WMA (n=7)*	*Ref*	*ST5*	*WMA (n=6)*	*Ref*
	Navajo (n=108)	**2.2 (1.5 – 3.6)**		*Navajo (n=42)*	**2.3 (1.6 – 3.8)**
CC8	*WMA (n=51)*	*Ref*	*ST8*	*WMA (n=47)*	*Ref*
				Navajo (n=129)	**0.5 (0.4 – 0.6)****
	Navajo (n=175)	**0.5 (0.4 – 0.6)****	*ST72*	*WMA (n=2)*	*Ref*
				Navajo (n=23)	2.0 (0.9 – 5.0)
CC30	*WMA (n=2)*	*Ref*	*ST30*	*WMA (n=1)*	*Ref*
	Navajo (n=64)	1.2 (0.4– 4.0)		*Navajo (n=14)*	2.3 (0.6 – 8.0)
CC45	*WMA (n=2)*	*Ref*	*ST45*	*WMA (n=2)*	*Ref*
	Navajo (n=23)	1.7 (0.7 – 4.5)		*Navajo (n=20)*	1.7 (0.7 – 4.3)
CC97	*WMA (n=2)*	*Ref*	*ST97*	*WMA (n=2)*	*Ref*
	Navajo (n=51)	**3.8 (1.7 – 10.0)***		*Navajo (n=46)*	**4.0 (1.8 – 10.0)**

BPI: bootstrap percentile interval; Ref: reference. Fisher test: * p<0.05; ** p<0.01

Supplemental Table 3. Comparison of clonal complex (CC) and sequence type (ST) frequencies between isolates from carriage (n=125) and invasive disease (n=195) in the Navajo Nation and White Mountain Apache Tribal lands, 2017

Clonal Complex	Collection source	Relative Frequency Ratio (95%BPI)	ST	Collection source	Relative Frequency Ratio (95%BPI)
CC1	*Carriage (n=28)*	*Ref*	*ST1*	*Carriage (n=4)*	*Ref*
				Invasive (n=10)	1.4 (0.8 - 3.0)
	Invasive (n=39)	0.9 (0.6 - 1.2)	*ST188*	*Carriage(n=23)*	*Ref*
				Invasive (n=56)	**0.6 (0.5 – 0.9)**
CC5	*Carriage (n=27)*	*Ref*	*ST5*	*Carriage(n=24)*	*Ref*
	Invasive (n=34)	0.8 (0.6 - 1.1)		*Invasive (n=28)*	**0.7 (0.5 – 0.9)**
CC8	*Carriage (n=22)*	*Ref*	*ST8*	*Carriage (n=9)*	*Ref*
				Invasive (n=56)	**3.7 (2.7 - 5.6)****
	Invasive (n=81)	**2.3 (1.8 - 3.1)****	*ST72*	*Carriage(n=13)*	*Ref*
				Invasive (n=16)	0.7 (0.5 - 1.1)
CC30	*Carriage(n=21)*	*Ref*	*ST30*	*Carriage(n=6)*	-
	Invasive (n=3)	**0.1 (0.0– 0.2)****		*Invasive (n=2)*	-
CC97	*Carriage (n=3)*	*Ref*	*ST97*	*Carriage (n=3)*	*Ref*
	Invasive (n=13)	**2.8 (1.3 – 7.7)**		*Invasive (n=11)*	**2.2 (1.1 – 5.7)**

BPI: bootstrap percentile interval; Ref: reference. Fisher test: * p<0.05; ** p<0.01

Supplemental Table 4. Comparison of clonal complex (CC) and sequence type (ST) frequencies in isolates from cases of bacteremia between study populations: Navajo Nation and White Mountain Apache Tribal lands (N/WMA) (n=376) and New Hampshire (NH) (n=189), 2016-2018

Clonal Complex	Population	Relative Frequency Ratio (95%BPI)	ST	Population	Relative Frequency Ratio (95%BPI)
CC1	*NH (n=10)*	*Ref*	ST1	*NH (n=4)*	*Ref*
				N/WMA (n=14)	1.7 (1.3 – 5.0)
	N/WMA(n=57)	**2.8 (1.7 - 5.0)****	*ST188*	*NH (n=4)*	*Ref*
				N/WMA (n=38)	**4.7 (2.3 - 12.0)****
CC5	*NH (n=50)*	*Ref*	ST5	*NH (n=34)*	*Ref*
				N/WMA (n=51)	**0.7 (0.5 – 1.0)**
	N/WMA (n=65)	**0.6 (0.5 – 0.9)***	ST105	*NH (n=11)*	*Ref*
				N/WMA (n=5)	**0.2 (0.1 – 1.0)****
CC8	*NH (n=55)*	*Ref*	*ST8*	*NH (n=41)*	*Ref*
				N/WMA (n=122)	**1.4 (1.1 - 1.8)****
	N/WMA (n=157)	**1.4 (1.1 - 1.7)****	*ST72*	*NH (n=10)*	*Ref*
				N/WMA (n=19)	1.2 (0.6 – 2.2)
CC30	*NH (n=23)*	*Ref*	*ST30*	*NH (n=18)*	*Ref*
	N/WMA (n=8)	**0.2 (0.0 – 0.4)****		*N/WMA (n=6)*	**0.2 (0.0 – 0.4)****
CC45	*NH (n=16)*	*Ref*	*ST45*	*NH (n=11)*	*Ref*
	N/WMA (n=15)	**0.5 (0.2 – 0.9)***		*N/WMA (n=15)*	0.6 (0.3 -1.3)
CC97	*NH (n=7)*	*Ref*	ST97	*NH (n=7)*	*Ref*
	N/WMA (n=35)	**2.4 (1.3 - 5.4)***		*N/WMA (n=32)*	**2.1 (1.2 - 4.3)***

BPI: bootstrap percentile interval; Ref: reference. Fisher test: * p<0.05; ** p<0.01

Supplemental Figure 1. Proportion of *Staphylococcus aureus* strains from invasive disease isolates from N/WMA Tribal lands that were methicillin resistant by A) collection year and B) collection year, clonal complex (CC) stratified by methicillin-susceptibility (MSSA) and resistance (MRSA).

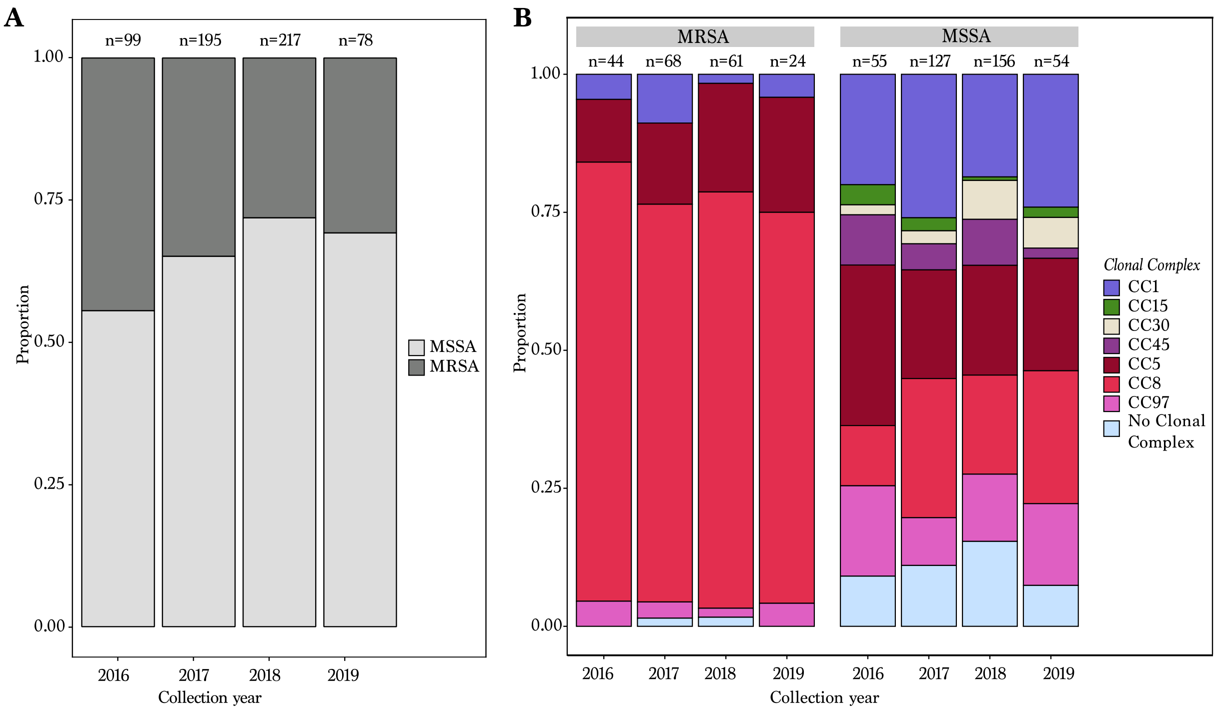

Supplemental File 1. Accession numbers for carriage and invasive isolates

ID	source	MLST	CC	year	Methicillin susceptibility/resistance	sccmec	Biosample.zaccession	Reads.accession
AN_003_G1	carriage	188	CC1	2017	MSSA	-	SAMN15567907	SRR12343931 (S); SRR12343857 (L)
AN_005_G1	carriage	51	NoComplex	2017	MSSA	-	SAMN15567908	SRR12343931 (S); SRR12343857 (L)
AN_009_G1	carriage	8	CC8	2017	MSSA	-	SAMN15567909	SRR12343939 (S); SRR12343855 (L)
AN_016_G1	carriage	97	CC97	2017	MSSA	-	SAMN15567910	SRR12343931 (S); SRR12343857 (L)
AN_023_G1	carriage	72	CC8	2017	MSSA	-	SAMN15567911	SRR12343931 (S); SRR12343857 (L)
AN_030_G1	carriage	5	CC5	2017	MSSA	-	SAMN15567912	SRR12343902 (S); SRR12343852 (L)
AN_036_G1	carriage	188	CC1	2017	MSSA	-	SAMN15567913	SRR12343931 (S); SRR12343857 (L)
AN_044_G1	carriage	5	CC5	2017	MSSA	-	SAMN15567914	SRR12343880 (S); SRR12343850 (L)
AN_047_G1	carriage	72	CC8	2017	MSSA	-	SAMN15567915	SRR12343869 (S); SRR12343849 (L)
AN_050_G1	carriage	45	CC45	2017	MSSA	-	SAMN15567916	SRR12343931 (S); SRR12343857 (L)
AN_053_G1	carriage	30	CC30	2017	MSSA	-	SAMN35040342	SRR24507891 (S); SRR24507863 (L)
AN_068_G1	carriage	6956	NoComplex	2017	MSSA	-	SAMN15567918	SRR12343931 (S); SRR12343857 (L)
AN_076_G1	carriage	51	NoComplex	2017	MSSA	-	SAMN15567919	SRR12344018 (S); SRR12344027 (L)
AN_079_G1	carriage	5	CC5	2017	MSSA	-	SAMN15567920	SRR12343931 (S); SRR12343857 (L)
AN_085_G1	carriage	188	CC1	2017	MSSA	-	SAMN15567921	SRR12343996 (S); SRR12344025 (L)
AN_099_G1	carriage	6	CC5	2017	MSSA	-	SAMN15567923	SRR12343931 (S); SRR12343857 (L)
AN_101_G1	carriage	582	CC15	2017	MSSA	-	SAMN15567925	SRR12343974 (S); SRR12344023 (L)
AN_102_G1	carriage	1	CC1	2017	MRSA	IVa(2B)	SAMN15567926	SRR12343963 (S); SRR12344022 (L)
AN_109_G1	carriage	72	CC8	2017	MSSA	-	SAMN15567927	SRR12343931 (S); SRR12343857 (L)
AN_111_G1	carriage	5	CC5	2017	MSSA	-	SAMN15567928	SRR12343931 (S); SRR12343857 (L)
AN_115_G1	carriage	5	CC5	2017	MSSA	-	SAMN15567929	SRR12343931 (S); SRR12343857 (L)
AN_123_G1	carriage	8	CC8	2017	MRSA	IVa(2B)	SAMN15567930	SRR12343931 (S); SRR12343857 (L)
AN_130_G1	carriage	59	NoComplex	2017	MSSA	-	SAMN15567931	SRR12343931 (S); SRR12343857 (L)
AN_136_G1	carriage	97	CC97	2017	MSSA	-	SAMN15567932	SRR12343931 (S); SRR12343857 (L)
AN_141_G1	carriage	30	CC30	2017	MSSA	-	SAMN15567933	SRR12343931 (S); SRR12343857 (L)
AN_142_G1	carriage	7314	CC30	2017	MSSA	-	SAMN15567934	SRR12343931 (S); SRR12343857 (L)
AN_143_G1	carriage	5	CC5	2017	MSSA	-	SAMN15567935	SRR12343931 (S); SRR12343857 (L)
AN_149_G1	carriage	72	CC8	2017	MSSA	-	SAMN15567936	SRR12343932 (S); SRR12344011 (L)
AN_150_G1	carriage	30	CC30	2017	MSSA	-	SAMN15567937	SRR12343931 (S); SRR12343857 (L)
AN_152_G1	carriage	72	CC8	2017	MSSA	-	SAMN15567938	SRR12343926 (S); SRR12344009 (L)
AN_157_G1	carriage	182	NoComplex	2017	MSSA	-	SAMN15567939	SRR12343925 (S); SRR12344008 (L)
AN_175_G1	carriage	72	CC8	2017	MSSA	-	SAMN15567940	SRR12343923 (S); SRR12344006 (L)
AN_176_G1	carriage	5	CC5	2017	MSSA	-	SAMN15567941	SRR12343931 (S); SRR12343857 (L)
AN_181_G1	carriage	72	CC8	2017	MSSA	-	SAMN15567942	SRR12343921 (S); SRR12344004 (L)
AN_184_G1	carriage	5	CC5	2017	MSSA	-	SAMN15567943	SRR12343931 (S); SRR12343857 (L)
AN_203_G1	carriage	5	CC5	2017	MSSA	-	SAMN15567944	SRR12343931 (S); SRR12343857 (L)

AN_204_G1	carriage	5	CC5	2017	MSSA	-	SAMN15567945	SRR12343931 (S); SRR12343857 (L)
AN_205_G1	carriage	5	CC5	2017	MSSA	-	SAMN15567946	SRR12343931 (S); SRR12343857 (L)
AN_206_G1	carriage	188	CC1	2017	MSSA	-	SAMN15567947	SRR12343931 (S); SRR12343857 (L)
AN_208_G1	carriage	30	CC30	2017	MSSA	-	SAMN15567948	SRR12343915 (S); SRR12343998 (L)
AN_212_G1	carriage	6176	NoComplex	2017	MSSA	-	SAMN15567949	SRR12343914 (S); SRR12343997 (L)
AN_225_G1	carriage	6176	NoComplex	2017	MSSA	-	SAMN35040381	SRR24507800
AN_230_G1	carriage	7565	NoComplex	2017	MSSA	-	SAMN35040384	SRR24507797
AN_233_G1	carriage	5	CC5	2017	MSSA	-	SAMN15567950	SRR12343912 (S); SRR12343995 (L)
AN_239_G1	carriage	5	CC5	2017	MSSA	-	SAMN15567951	SRR12343931 (S); SRR12343857 (L)
AN_247_G1	carriage	72	CC8	2017	MRSA	IVa(2B)	SAMN15567952	SRR12343931 (S); SRR12343857 (L)
AN_250_G1	carriage	9	CC1	2017	MSSA	-	SAMN15567953	SRR12343931 (S); SRR12343857 (L)
AN_258_G1	carriage	8	CC8	2017	MRSA	IVa(2B)	SAMN15567954	SRR12343908 (S); SRR12343991 (L)
AN_260_G1	carriage	1	CC1	2017	MRSA	IVa(2B)	SAMN15567955	SRR12343931 (S); SRR12343857 (L)
AN_260_G2	carriage	188	CC1	2017	MSSA	-	SAMN35040392	SRR24507788
AN_261_G1	carriage	51	NoComplex	2017	MSSA	-	SAMN15567956	SRR12343931 (S); SRR12343857 (L)
AN_265_G1	carriage	25	NoComplex	2017	MSSA	-	SAMN15567957	SRR12343931 (S); SRR12343857 (L)
AN_270_G1	carriage	188	CC1	2017	MSSA	-	SAMN15567958	SRR12343931 (S); SRR12343857 (L)
AN_275_G1	carriage	188	CC1	2017	MSSA	-	SAMN15567959	SRR12343903 (S); SRR12343986 (L)
AN_285_G1	carriage	8	CC8	2017	MSSA	-	SAMN15567960	SRR12343901 (S); SRR12343984 (L)
AN_286_G1	carriage	188	CC1	2017	MSSA	-	SAMN15567961	SRR12343931 (S); SRR12343857 (L)
BDWV0N	invasive disease	20	NoComplex	2017	MSSA	-	SAMN49528396	SRR34096774
BDWV0R	invasive disease	188	CC1	2017	MSSA	-	SAMN49528397	SRR34096773
BDWV0S	invasive disease	8	CC8	2017	MSSA	-	SAMN49528398	SRR34096469
BDWV0T	invasive disease	97	CC97	2017	MRSA	IVa	SAMN49528399	SRR34096574
BDWV0V	invasive disease	1	CC1	2017	MRSA	IV(2B)	SAMN49528400	SRR34096535
BDWV0X	invasive disease	25	NoComplex	2017	MSSA	-	SAMN49528401	SRR34096653
BDWV0Y	invasive disease	5	CC5	2017	MSSA	-	SAMN49528402	SRR34096805
BDWV0Z	invasive disease	8	CC8	2017	MRSA	IVa	SAMN49528403	SRR34096794
BDWV10	invasive disease	8	CC8	2017	MSSA	-	SAMN49528404	SRR34096783
BDWV11	invasive disease	6357	CC15	2017	MSSA	-	SAMN49528405	SRR34096628
BDWV12	invasive disease	87	NoComplex	2017	MSSA	-	SAMN49528406	SRR34096772
BDWV13	invasive disease	8	CC8	2017	MRSA	IVa	SAMN49528407	SRR34096761
BDWV14	invasive disease	97	CC97	2017	MSSA	-	SAMN49528408	SRR34096750
BDWV15	invasive disease	97	CC97	2017	MSSA	-	SAMN49528409	SRR34096739
BDWV16	invasive disease	3465	CC8	2017	MRSA	IVa	SAMN49528410	SRR34096728
BDWV17	invasive disease	6358	CC8	2017	MSSA	-	SAMN49528411	SRR34096717
BDWV18	invasive disease	1	CC1	2017	MRSA	IVa	SAMN49528412	SRR34096706
BDWV19	invasive disease	5	CC5	2017	MRSA	IIa	SAMN49528413	SRR34096610
BDWV1B	invasive disease	109	CC1	2017	MSSA	-	SAMN49528414	SRR34096599

BDWV1C	invasive disease	8	CC8	2017	MRSA	IV(2B)	SAMN49528415	SRR34096588
BDWV1D	invasive disease	72	CC8	2017	MSSA	-	SAMN49528416	SRR34096468
BDWV1F	invasive disease	398	NoComplex	2017	MSSA	-	SAMN49528417	SRR34096457
BDWV1G	invasive disease	101	NoComplex	2018	MSSA	-	SAMN49528418	SRR34096446
BDWV1H	invasive disease	9	CC1	2018	MSSA	-	SAMN49528419	SRR34096916
BDWV1J	invasive disease	45	CC45	2018	MSSA	-	SAMN49528420	SRR34096905
BDWV1K	invasive disease	8	CC8	2018	MRSA	IVa	SAMN49528421	SRR34096894
BDWV1L	invasive disease	6177	CC5	2018	MRSA	IIa	SAMN49528422	SRR34096883
BDWV1M	invasive disease	8	CC8	2018	MRSA	IVa	SAMN49528423	SRR34096692
BDWV1N	invasive disease	87	NoComplex	2018	MSSA	-	SAMN49528424	SRR34096681
BDWV1P	invasive disease	97	CC97	2018	MSSA	-	SAMN49528425	SRR34096670
BDWV1R	invasive disease	188	CC1	2018	MSSA	-	SAMN49528426	SRR34096573
BDWV1V	invasive disease	12	NoComplex	2018	MSSA	-	SAMN49528427	SRR34096562
BDWV1W	invasive disease	8	CC8	2018	MRSA	IVa	SAMN49528428	SRR34096551
BDWV1X	invasive disease	8	CC8	2018	MRSA	IVa	SAMN49528429	SRR34097021
BDWV1Y	invasive disease	8	CC8	2018	MRSA	IVa	SAMN49528430	SRR34097010
BDWV1Z	invasive disease	72	CC8	2018	MSSA	-	SAMN49528431	SRR34096999
BDWV20	invasive disease	5	CC5	2018	MSSA	-	SAMN49528432	SRR34096880
BDWV21	invasive disease	1	CC1	2018	MSSA	-	SAMN49528433	SRR34096869
BDWV22	invasive disease	6359	CC97	2018	MSSA	-	SAMN49528434	SRR34096858
BDWV23	invasive disease	5	CC5	2018	MSSA	-	SAMN49528435	SRR34096847
BDWV24	invasive disease	5	CC5	2018	MSSA	-	SAMN49528436	SRR34096534
BDWV25	invasive disease	45	CC45	2018	MSSA	-	SAMN49528437	SRR34096523
BDWV27	invasive disease	8	CC8	2018	MRSA	IVa	SAMN49528438	SRR34096512
BDWV29	invasive disease	5	CC5	2018	MSSA	-	SAMN49528439	SRR34096982
BDWV2C	invasive disease	97	CC97	2018	MSSA	-	SAMN49528440	SRR34096971
BDWV2D	invasive disease	188	CC1	2018	MSSA	-	SAMN49528441	SRR34096960
BDWV2F	invasive disease	8	CC8	2018	MRSA	IVa	SAMN49528442	SRR34096841
BDWV2G	invasive disease	950	CC5	2018	MSSA	-	SAMN49528443	SRR34096830
BDWV2H	invasive disease	72	CC8	2018	MSSA	-	SAMN49528444	SRR34096819
BDWV2K	invasive disease	8	CC8	2018	MRSA	IVa	SAMN49528445	SRR34096664
BDWV2L	invasive disease	8	CC8	2018	MRSA	IVa	SAMN49528446	SRR34096652
BDWV2M	invasive disease	8	CC8	2018	MRSA	IVa	SAMN49528447	SRR34096641
BDWV2P	invasive disease	8	CC8	2018	MSSA	-	SAMN49528448	SRR34096509
BDWV2R	invasive disease	5	CC5	2018	MRSA	IVa	SAMN49528449	SRR34096498
BDWV2S	invasive disease	97	CC97	2018	MSSA	-	SAMN49528450	SRR34096487
BDWV2T	invasive disease	97	CC97	2018	MSSA	-	SAMN49528451	SRR34096476
BDWV2W	invasive disease	5	CC5	2018	MSSA	-	SAMN49528452	SRR34096946
BDWV2X	invasive disease	5	CC5	2018	MSSA	-	SAMN49528453	SRR34096935

BDWV2Y	invasive disease	105	CC5	2018	MRSA	IIa	SAMN49528454	SRR34096924
BDWV2Z	invasive disease	8	CC8	2018	MRSA	IVa	SAMN49528455	SRR34096806
BDWV31	invasive disease	6361	CC45	2018	MSSA	-	SAMN49528456	SRR34096804
BDWV32	invasive disease	8	CC8	2018	MRSA	IVa	SAMN49528457	SRR34096803
BDWV34	invasive disease	1159	CC8	2018	MSSA	-	SAMN49528458	SRR34096802
BDWV35	invasive disease	398	NoComplex	2018	MSSA	-	SAMN49528459	SRR34096801
BDWV36	invasive disease	8	CC8	2018	MSSA	-	SAMN49528460	SRR34096800
BDWV37	invasive disease	105	CC5	2018	MRSA	IIa	SAMN49528461	SRR34096799
BDWV39	invasive disease	1181	CC8	2018	MSSA	-	SAMN49528462	SRR34096798
BDWV3B	invasive disease	97	CC97	2018	MSSA	-	SAMN49528463	SRR34096797
BDWV3F	invasive disease	6362	NoComplex	2018	MSSA	-	SAMN49528464	SRR34096796
BDWV3G	invasive disease	5	CC5	2018	MSSA	-	SAMN49528465	SRR34096795
BDWV3H	invasive disease	72	CC8	2018	MSSA	-	SAMN49528466	SRR34096793
BDWV3J	invasive disease	5	CC5	2018	MSSA	-	SAMN49528467	SRR34096792
BDWV3K	invasive disease	5	CC5	2018	MSSA	-	SAMN49528468	SRR34096791
BDWV3L	invasive disease	45	CC45	2018	MSSA	-	SAMN49528469	SRR34096790
BDWV3M	invasive disease	5	CC5	2018	MSSA	-	SAMN49528470	SRR34096789
BDWV3N	invasive disease	97	CC97	2018	MSSA	-	SAMN49528471	SRR34096788
BDWV3P	invasive disease	1	CC1	2018	MRSA	IVa	SAMN49528472	SRR34096787
BDWV3R	invasive disease	5	CC5	2018	MSSA	-	SAMN49528473	SRR34096786
BDWV3S	invasive disease	8	CC8	2018	MRSA	IVa	SAMN49528474	SRR34096785
BDWV3T	invasive disease	5	CC5	2018	MRSA	IIa	SAMN49528475	SRR34096784
BDWV3V	invasive disease	30	CC30	2018	MSSA	-	SAMN49528476	SRR34096782
BDWV3W	invasive disease	8	CC8	2018	MRSA	IVa	SAMN49528477	SRR34096781
BDWV3X	invasive disease	188	CC1	2018	MSSA	-	SAMN49528478	SRR34096780
BDWV3Y	invasive disease	188	CC1	2018	MSSA	-	SAMN49528479	SRR34096779
BDWV41	invasive disease	20	NoComplex	2018	MSSA	-	SAMN49528480	SRR34096778
BDWV42	invasive disease	25	NoComplex	2018	MSSA	-	SAMN49528481	SRR34096777
BDWV43	invasive disease	97	CC97	2018	MSSA	-	SAMN49528482	SRR34096776
BDWV45	invasive disease	8	CC8	2018	MSSA	-	SAMN49528483	SRR34096775
BDWV46	invasive disease	12	NoComplex	2018	MSSA	-	SAMN49528484	SRR34096630
BDWV47	invasive disease	5	CC5	2018	MSSA	-	SAMN49528485	SRR34096629
BDWV48	invasive disease	6375	CC8	2018	MRSA	IVa	SAMN49528486	SRR34096627
BDWV4B	invasive disease	8	CC8	2018	MRSA	IVa	SAMN49528487	SRR34096626
BDWV4C	invasive disease	6363	CC5	2018	MSSA	-	SAMN49528488	SRR34096625
BDWV4D	invasive disease	5	CC5	2018	MSSA	-	SAMN49528489	SRR34096624
BDWV4F	invasive disease	6364	CC8	2018	MSSA	-	SAMN49528490	SRR34096623
BDWV4G	invasive disease	6364	CC8	2018	MSSA	-	SAMN49528491	SRR34096622
BDWV4H	invasive disease	8	CC8	2018	MRSA	IVa	SAMN49528492	SRR34096621

BDWV4J	invasive disease	8	CC8	2018	MRSA	IVa	SAMN49528493	SRR34096620
BDWV4K	invasive disease	8	CC8	2018	MSSA	-	SAMN49528494	SRR34096619
BDWV4L	invasive disease	8	CC8	2018	MRSA	IVa	SAMN49528495	SRR34096618
BDWV4M	invasive disease	188	CC1	2018	MSSA	-	SAMN49528496	SRR34096771
BDWV4N	invasive disease	8	CC8	2018	MSSA	-	SAMN49528497	SRR34096770
BDWV4W	invasive disease	6177	CC5	2018	MRSA	IIa	SAMN49528498	SRR34096769
BDWV4Y	invasive disease	45	CC45	2018	MSSA	-	SAMN49528499	SRR34096768
BDWV4Z	invasive disease	5	CC5	2018	MSSA	-	SAMN49528500	SRR34096767
BDWV50	invasive disease	45	CC45	2018	MSSA	-	SAMN49528501	SRR34096766
BDWV51	invasive disease	5	CC5	2018	MSSA	-	SAMN49528502	SRR34096765
BDWV52	invasive disease	188	CC1	2018	MSSA	-	SAMN49528503	SRR34096764
BDWV53	invasive disease	6365	CC45	2018	MSSA	-	SAMN49528504	SRR34096763
BDWV54	invasive disease	97	CC97	2018	MSSA	-	SAMN49528505	SRR34096762
BDWV55	invasive disease	25	NoComplex	2018	MSSA	-	SAMN49528506	SRR34096760
BDWV56	invasive disease	188	CC1	2018	MSSA	-	SAMN49528507	SRR34096759
BDWV57	invasive disease	8	CC8	2018	MRSA	IVa	SAMN49528508	SRR34096758
BDWV58	invasive disease	6366	CC5	2018	MSSA	-	SAMN49528509	SRR34096757
BDWV59	invasive disease	5	CC5	2018	MRSA	IIa	SAMN49528510	SRR34096756
BDWV5B	invasive disease	6177	CC5	2018	MRSA	IIa	SAMN49528511	SRR34096755
BDWV5D	invasive disease	97	CC97	2018	MSSA	-	SAMN49528512	SRR34096754
BDWV5F	invasive disease	45	CC45	2018	MSSA	-	SAMN49528513	SRR34096753
BDWV5G	invasive disease	8	CC8	2018	MRSA	IVa	SAMN49528514	SRR34096752
BDWV5H	invasive disease	8	CC8	2018	MRSA	IVa	SAMN49528515	SRR34096751
BDWV5J	invasive disease	97	CC97	2018	MRSA	IVa	SAMN49528516	SRR34096749
BDWV5K	invasive disease	unknownST	NoComplex	2018	MSSA	-	SAMN49528517	SRR34096748
BDWV5L	invasive disease	15	CC15	2018	MSSA	-	SAMN49528518	SRR34096747
BDWV5M	invasive disease	8	CC8	2018	MRSA	IVa	SAMN49528519	SRR34096746
BDWV5N	invasive disease	5	CC5	2018	MSSA	-	SAMN49528520	SRR34096745
BDWV5P	invasive disease	8	CC8	2018	MRSA	IVa	SAMN49528521	SRR34096744
BDWV5R	invasive disease	5	CC5	2018	MRSA	IIa	SAMN49528522	SRR34096743
BDWV5S	invasive disease	6367	NoComplex	2018	MSSA	-	SAMN49528523	SRR34096742
BDWV5T	invasive disease	45	CC45	2018	MSSA	-	SAMN49528524	SRR34096741
BDWV5V	invasive disease	5	CC5	2018	MSSA	-	SAMN49528525	SRR34096740
BDWV5W	invasive disease	188	CC1	2018	MSSA	-	SAMN49528526	SRR34096738
BDWV5X	invasive disease	97	CC97	2018	MSSA	-	SAMN49528527	SRR34096737
BDWV5Y	invasive disease	8	CC8	2018	MSSA	-	SAMN49528528	SRR34096736
BDWV5Z	invasive disease	6368	CC1	2018	MSSA	-	SAMN49528529	SRR34096735
BDWV60	invasive disease	6369	CC8	2018	MSSA	-	SAMN49528530	SRR34096734
BDWV62	invasive disease	30	CC30	2018	MSSA	-	SAMN49528531	SRR34096733

BDWV63	invasive disease	88	NoComplex	2018	MSSA	-	SAMN49528532	SRR34096732
BDWV64	invasive disease	188	CC1	2018	MSSA	-	SAMN49528533	SRR34096731
BDWV65	invasive disease	188	CC1	2018	MSSA	-	SAMN49528534	SRR34096730
BDWV66	invasive disease	12	NoComplex	2018	MSSA	-	SAMN49528535	SRR34096729
BDWV67	invasive disease	97	CC97	2018	MSSA	-	SAMN49528536	SRR34096727
BDWV68	invasive disease	5	CC5	2018	MSSA	-	SAMN49528537	SRR34096726
BDWV6B	invasive disease	5	CC5	2018	MSSA	-	SAMN49528538	SRR34096725
BDWV6D	invasive disease	30	CC30	2018	MSSA	-	SAMN49528539	SRR34096724
BDWV6F	invasive disease	5	CC5	2018	MSSA	-	SAMN49528540	SRR34096723
BDWV6G	invasive disease	5	CC5	2018	MRSA	IIa	SAMN49528541	SRR34096722
BDWV6H	invasive disease	188	CC1	2018	MSSA	-	SAMN49528542	SRR34096721
BDWV6K	invasive disease	6370	CC8	2018	MRSA	IVa	SAMN49528543	SRR34096720
BDWV6L	invasive disease	20	NoComplex	2018	MSSA	-	SAMN49528544	SRR34096719
BDWV6M	invasive disease	188	CC1	2018	MSSA	-	SAMN49528545	SRR34096718
BDWV6N	invasive disease	45	CC45	2018	MSSA	-	SAMN49528546	SRR34096716
BDWV6S	invasive disease	59	NoComplex	2018	MSSA	-	SAMN49528547	SRR34096715
BDWV6T	invasive disease	8	CC8	2018	MRSA	IVa	SAMN49528548	SRR34096714
BDWV6V	invasive disease	8	CC8	2018	MRSA	IVa	SAMN49528549	SRR34096713
BDWV6W	invasive disease	5	CC5	2018	MSSA	-	SAMN49528550	SRR34096712
BDWV6X	invasive disease	59	NoComplex	2018	MSSA	-	SAMN49528551	SRR34096711
BDWV6Y	invasive disease	97	CC97	2018	MSSA	-	SAMN49528552	SRR34096710
BDWV6Z	invasive disease	5	CC5	2018	MSSA	-	SAMN49528553	SRR34096709
BDWV70	invasive disease	8	CC8	2018	MSSA	-	SAMN49528554	SRR34096708
BDWV71	invasive disease	2884	NoComplex	2018	MSSA	-	SAMN49528555	SRR34096707
BDWV73	invasive disease	8	CC8	2018	MSSA	-	SAMN49528556	SRR34096705
BDWV74	invasive disease	45	CC45	2018	MSSA	-	SAMN49528557	SRR34096704
BDWV75	invasive disease	8	CC8	2018	MRSA	IVa	SAMN49528558	SRR34096703
BDWV76	invasive disease	8	CC8	2018	MSSA	-	SAMN49528559	SRR34096617
BDWV77	invasive disease	6371	CC30	2018	MSSA	-	SAMN49528560	SRR34096616
BDWV7B	invasive disease	5	CC5	2018	MSSA	-	SAMN49528561	SRR34096615
BDWV7D	invasive disease	6373	CC5	2018	MSSA	-	SAMN49528562	SRR34096614
BDWV7F	invasive disease	8	CC8	2018	MRSA	IVa	SAMN49528563	SRR34096613
BDWV7H	invasive disease	6372	CC8	2018	MRSA	IVa	SAMN49528564	SRR34096612
BDWV7J	invasive disease	97	CC97	2018	MSSA	-	SAMN49528565	SRR34096611
BDWV7K	invasive disease	8	CC8	2018	MSSA	-	SAMN49528566	SRR34096609
BDWV7L	invasive disease	188	CC1	2018	MSSA	-	SAMN49528567	SRR34096608
BDWV7P	invasive disease	188	CC1	2018	MSSA	-	SAMN49528568	SRR34096607
BDWV7R	invasive disease	30	CC30	2018	MSSA	-	SAMN49528569	SRR34096606
BDWV7S	invasive disease	8	CC8	2018	MRSA	IVa	SAMN49528570	SRR34096605

BDWV7T	invasive disease	8	CC8	2018	MSSA	-	SAMN49528571	SRR34096604
BDWV7V	invasive disease	6374	CC5	2018	MSSA	-	SAMN49528572	SRR34096603
BDWV7W	invasive disease	8	CC8	2018	MRSA	IVa	SAMN49528573	SRR34096602
BDWV7X	invasive disease	8	CC8	2018	MRSA	IVa	SAMN49528574	SRR34096601
BDWV7Y	invasive disease	188	CC1	2018	MSSA	-	SAMN49528575	SRR34096600
BDWV7Z	invasive disease	188	CC1	2018	MSSA	-	SAMN49528576	SRR34096598
BDWV80	invasive disease	188	CC1	2018	MSSA	-	SAMN49528577	SRR34096597
BDWV82	invasive disease	630	CC8	2018	MSSA	-	SAMN49528578	SRR34096596
BDWV83	invasive disease	88	NoComplex	2018	MSSA	-	SAMN49528579	SRR34096595
BDWV84	invasive disease	30	CC30	2018	MSSA	-	SAMN49528580	SRR34096594
BDWV85	invasive disease	20	NoComplex	2018	MSSA	-	SAMN49528581	SRR34096593
BDWV86	invasive disease	8	CC8	2018	MRSA	IVa	SAMN49528582	SRR34096592
BDWV88	invasive disease	8	CC8	2018	MRSA	IVa	SAMN49528583	SRR34096591
BDWV89	invasive disease	1	CC1	2018	MSSA	-	SAMN49528584	SRR34096590
BDWV8B	invasive disease	30	CC30	2018	MSSA	-	SAMN49528585	SRR34096589
BDWV8C	invasive disease	87	NoComplex	2018	MSSA	-	SAMN49528586	SRR34096587
BDWV8D	invasive disease	8	CC8	2018	MRSA	IVa	SAMN49528587	SRR34096586
BDWV8F	invasive disease	45	CC45	2018	MSSA	-	SAMN49528588	SRR34096585
BDWV8G	invasive disease	97	CC97	2018	MSSA	-	SAMN49528589	SRR34096584
BDWV8H	invasive disease	6375	CC8	2018	MSSA	-	SAMN49528590	SRR34096583
BDWV8J	invasive disease	5	CC5	2018	MRSA	IIa	SAMN49528591	SRR34096582
BDWV8K	invasive disease	45	CC45	2018	MSSA	-	SAMN49528592	SRR34096473
BDWV8L	invasive disease	188	CC1	2018	MSSA	-	SAMN49528593	SRR34096472
BDWV8M	invasive disease	188	CC1	2018	MSSA	-	SAMN49528594	SRR34096471
BFJM6K	invasive disease	5	CC5	2017	MSSA	-	SAMN49528595	SRR34096470
BFJM6L	invasive disease	72	CC8	2017	MSSA	-	SAMN49528596	SRR34096467
BFJM6M	invasive disease	8	CC8	2017	MSSA	-	SAMN49528597	SRR34096466
BFJM6N	invasive disease	188	CC1	2017	MSSA	-	SAMN49528598	SRR34096465
BGB515	invasive disease	8	CC8	2016	MRSA	IVa	SAMN49528599	SRR34096464
BGB516	invasive disease	8	CC8	2016	MRSA	IVa	SAMN49528600	SRR34096463
BGB517	invasive disease	20	NoComplex	2016	MSSA	-	SAMN49528601	SRR34096462
BGB518	invasive disease	188	CC1	2016	MSSA	-	SAMN49528602	SRR34096461
BGB519	invasive disease	97	CC97	2016	MRSA	IVa	SAMN49528603	SRR34096460
BGB51C	invasive disease	5	CC5	2016	MSSA	-	SAMN49528604	SRR34096459
BGB51D	invasive disease	6376	CC97	2016	MSSA	-	SAMN49528605	SRR34096458
BGB51F	invasive disease	97	CC97	2016	MSSA	-	SAMN49528606	SRR34096456
BGB51G	invasive disease	188	CC1	2016	MSSA	-	SAMN49528607	SRR34096455
BGB51H	invasive disease	5	CC5	2016	MSSA	-	SAMN49528608	SRR34096454
BGB51J	invasive disease	188	CC1	2016	MSSA	-	SAMN49528609	SRR34096453

BGB51K	invasive disease	8	CC8	2016	MRSA	IVa	SAMN49528610	SRR34096452
BGB51N	invasive disease	8	CC8	2016	MRSA	IVa	SAMN49528611	SRR34096451
BGB51P	invasive disease	20	NoComplex	2016	MSSA	-	SAMN49528612	SRR34096450
BGB51R	invasive disease	5	CC5	2016	MSSA	-	SAMN49528613	SRR34096449
BGB51S	invasive disease	72	CC8	2016	MSSA	-	SAMN49528614	SRR34096448
BGB51T	invasive disease	8	CC8	2016	MRSA	IVa	SAMN49528615	SRR34096447
BGB51V	invasive disease	188	CC1	2016	MSSA	-	SAMN49528616	SRR34096445
BGB51W	invasive disease	5	CC5	2016	MRSA	IIa	SAMN49528617	SRR34096444
BGB51X	invasive disease	45	CC45	2016	MSSA	-	SAMN49528618	SRR34096443
BGB51Y	invasive disease	20	NoComplex	2016	MSSA	-	SAMN49528619	SRR34096442
BGB520	invasive disease	1	CC1	2016	MRSA	IVa	SAMN49528620	SRR34096441
BGB523	invasive disease	97	CC97	2016	MSSA	-	SAMN49528621	SRR34096440
BGB524	invasive disease	5	CC5	2016	MSSA	-	SAMN49528622	SRR34096439
BGB525	invasive disease	97	CC97	2016	MSSA	-	SAMN49528623	SRR34096438
BGB526	invasive disease	8	CC8	2016	MRSA	IVa	SAMN49528624	SRR34096918
BGB527	invasive disease	15	CC15	2016	MSSA	-	SAMN49528625	SRR34096917
BGB528	invasive disease	188	CC1	2016	MSSA	-	SAMN49528626	SRR34096915
BGB529	invasive disease	8	CC8	2016	MRSA	IVa	SAMN49528627	SRR34096914
BGB52D	invasive disease	8	CC8	2016	MRSA	IVa	SAMN49528628	SRR34096913
BGB52F	invasive disease	5	CC5	2016	MSSA	-	SAMN49528629	SRR34096912
BGB52G	invasive disease	8	CC8	2016	MRSA	IVa	SAMN49528630	SRR34096911
BGB52H	invasive disease	5	CC5	2016	MSSA	-	SAMN49528631	SRR34096910
BGB52J	invasive disease	105	CC5	2016	MRSA	IIa	SAMN49528632	SRR34096909
BGB52K	invasive disease	8	CC8	2016	MRSA	IVa	SAMN49528633	SRR34096908
BGB52M	invasive disease	6377	CC97	2016	MSSA	-	SAMN49528634	SRR34096907
BGB52N	invasive disease	6378	NoComplex	2016	MSSA	-	SAMN49528635	SRR34096906
BGB52P	invasive disease	8	CC8	2016	MRSA	IVa	SAMN49528636	SRR34096904
BGB52S	invasive disease	5	CC5	2016	MRSA	IIa	SAMN49528637	SRR34096903
BGB52T	invasive disease	8	CC8	2016	MRSA	IVa	SAMN49528638	SRR34096902
BGB52V	invasive disease	8	CC8	2016	MRSA	IVa	SAMN49528639	SRR34096901
BGB52W	invasive disease	4236	CC5	2016	MSSA	-	SAMN49528640	SRR34096900
BGB52X	invasive disease	8	CC8	2016	MRSA	IVa	SAMN49528641	SRR34096899
BGB52Y	invasive disease	1	CC1	2016	MRSA	IVa	SAMN49528642	SRR34096898
BGB52Z	invasive disease	5	CC5	2016	MSSA	-	SAMN49528643	SRR34096897
BGB530	invasive disease	8	CC8	2016	MRSA	IVa	SAMN49528644	SRR34096896
BGB531	invasive disease	8	CC8	2016	MSSA	-	SAMN49528645	SRR34096895
BGB532	invasive disease	8	CC8	2016	MRSA	IVa	SAMN49528646	SRR34096893
BGB533	invasive disease	188	CC1	2016	MSSA	-	SAMN49528647	SRR34096892
BGB534	invasive disease	1	CC1	2016	MSSA	-	SAMN49528648	SRR34096891

BGB536	invasive disease	25	NoComplex	2016	MSSA	-	SAMN49528649	SRR34096890
BGB537	invasive disease	5	CC5	2016	MSSA	-	SAMN49528650	SRR34096889
BGB539	invasive disease	8	CC8	2016	MRSA	IVa	SAMN49528651	SRR34096888
BGB53B	invasive disease	45	CC45	2016	MSSA	-	SAMN49528652	SRR34096887
BGB53C	invasive disease	8	CC8	2016	MRSA	IVa	SAMN49528653	SRR34096886
BGB53D	invasive disease	15	CC15	2016	MSSA	-	SAMN49528654	SRR34096885
BGB53F	invasive disease	188	CC1	2016	MSSA	-	SAMN49528655	SRR34096884
BGB53G	invasive disease	8	CC8	2016	MRSA	IVa	SAMN49528656	SRR34096702
BGB53H	invasive disease	8	CC8	2016	MRSA	IVa	SAMN49528657	SRR34096701
BGB53J	invasive disease	8	CC8	2016	MRSA	IVa	SAMN49528658	SRR34096700
BGB53L	invasive disease	5	CC5	2016	MSSA	-	SAMN49528659	SRR34096699
BGB53M	invasive disease	45	CC45	2016	MSSA	-	SAMN49528660	SRR34096698
BGB53N	invasive disease	45	CC45	2016	MSSA	-	SAMN49528661	SRR34096697
BGB53P	invasive disease	97	CC97	2016	MSSA	-	SAMN49528662	SRR34096696
BGB53S	invasive disease	5	CC5	2016	MRSA	IIa	SAMN49528663	SRR34096695
BGB53T	invasive disease	8	CC8	2016	MRSA	IVa	SAMN49528664	SRR34096694
BGB53V	invasive disease	5	CC5	2016	MSSA	-	SAMN49528665	SRR34096693
BGB53W	invasive disease	8	CC8	2016	MRSA	IVa	SAMN49528666	SRR34096691
BGB53Y	invasive disease	97	CC97	2016	MSSA	-	SAMN49528667	SRR34096690
BGB53Z	invasive disease	8	CC8	2016	MRSA	IVa	SAMN49528668	SRR34096689
BGB540	invasive disease	8	CC8	2016	MRSA	IVa	SAMN49528669	SRR34096688
BGB541	invasive disease	97	CC97	2016	MSSA	-	SAMN49528670	SRR34096687
BGB542	invasive disease	30	CC30	2016	MSSA	-	SAMN49528671	SRR34096686
BGB543	invasive disease	8	CC8	2016	MRSA	IVa	SAMN49528672	SRR34096685
BGB545	invasive disease	8	CC8	2016	MRSA	IVa	SAMN49528673	SRR34096684
BGB546	invasive disease	8	CC8	2016	MRSA	IVa	SAMN49528674	SRR34096683
BGB547	invasive disease	8	CC8	2016	MRSA	IVa	SAMN49528675	SRR34096682
BGB548	invasive disease	8	CC8	2016	MRSA	IVa	SAMN49528676	SRR34096680
BGB549	invasive disease	5	CC5	2016	MSSA	-	SAMN49528677	SRR34096679
BGB54B	invasive disease	8	CC8	2016	MSSA	-	SAMN49528678	SRR34096678
BGB54C	invasive disease	188	CC1	2016	MSSA	-	SAMN49528679	SRR34096677
BGB54D	invasive disease	45	CC45	2016	MSSA	-	SAMN49528680	SRR34096676
BGB54F	invasive disease	188	CC1	2016	MSSA	-	SAMN49528681	SRR34096675
BGB54G	invasive disease	8	CC8	2016	MRSA	IVa	SAMN49528682	SRR34096674
BGB54H	invasive disease	8	CC8	2016	MSSA	-	SAMN49528683	SRR34096673
BGB54J	invasive disease	5	CC5	2016	MSSA	-	SAMN49528684	SRR34096672
BGB54K	invasive disease	72	CC8	2016	MSSA	-	SAMN49528685	SRR34096671
BGB54M	invasive disease	8	CC8	2016	MSSA	-	SAMN49528686	SRR34096669
BGB54N	invasive disease	8	CC8	2016	MRSA	IVa	SAMN49528687	SRR34096668

BGB54P	invasive disease	8	CC8	2016	MRSA	IVa	SAMN49528688	SRR34096667
BGB54R	invasive disease	188	CC1	2016	MSSA	-	SAMN49528689	SRR34096581
BGB54T	invasive disease	8	CC8	2016	MRSA	IVa	SAMN49528690	SRR34096580
BGB54V	invasive disease	5	CC5	2016	MRSA	IIa	SAMN49528691	SRR34096579
BGB54W	invasive disease	5	CC5	2016	MSSA	-	SAMN49528692	SRR34096578
BGB54X	invasive disease	5	CC5	2016	MSSA	-	SAMN49528693	SRR34096577
BGB54Z	invasive disease	97	CC97	2016	MSSA	-	SAMN49528694	SRR34096576
BGB550	invasive disease	8	CC8	2016	MRSA	IVa	SAMN49528695	SRR34096575
BGB551	invasive disease	2932	CC97	2017	MSSA	-	SAMN49528696	SRR34096572
BGB552	invasive disease	5	CC5	2016	MSSA	-	SAMN49528697	SRR34096571
BGB554	invasive disease	6380	CC8	2017	MRSA	IVa	SAMN49528698	SRR34096570
BGB556	invasive disease	72	CC8	2017	MSSA	-	SAMN49528699	SRR34096569
BGB557	invasive disease	97	CC97	2017	MSSA	-	SAMN49528700	SRR34096568
BGB558	invasive disease	105	CC5	2017	MRSA	IIa	SAMN49528701	SRR34096567
BGB559	invasive disease	6381	CC5	2017	MSSA	-	SAMN49528702	SRR34096566
BGB55F	invasive disease	291	NoComplex	2017	MSSA	-	SAMN49528703	SRR34096565
BGB55G	invasive disease	8	CC8	2017	MRSA	IVa	SAMN49528704	SRR34096564
BGB55H	invasive disease	8	CC8	2017	MSSA	-	SAMN49528705	SRR34096563
BGB55J	invasive disease	8	CC8	2017	MRSA	IVa	SAMN49528706	SRR34096561
BGB55K	invasive disease	8	CC8	2017	MRSA	IVa	SAMN49528707	SRR34096560
BGB55P	invasive disease	12	NoComplex	2017	MSSA	-	SAMN49528708	SRR34096559
BGB55R	invasive disease	188	CC1	2017	MSSA	-	SAMN49528709	SRR34096558
BGB55T	invasive disease	789	CC8	2017	MSSA	-	SAMN49528710	SRR34096557
BGB55V	invasive disease	8	CC8	2017	MRSA	IVa	SAMN49528711	SRR34096556
BGB55W	invasive disease	5	CC5	2017	MSSA	-	SAMN49528712	SRR34096555
BGB55X	invasive disease	8	CC8	2017	MSSA	-	SAMN49528713	SRR34096554
BGB55Y	invasive disease	6382	CC30	2017	MSSA	-	SAMN49528714	SRR34096553
BGB55Z	invasive disease	97	CC97	2017	MSSA	-	SAMN49528715	SRR34096552
BGB560	invasive disease	6383	CC1	2017	MSSA	-	SAMN49528716	SRR34096550
BGB561	invasive disease	72	CC8	2017	MRSA	Vc	SAMN49528717	SRR34096549
BGB563	invasive disease	6384	CC8	2017	MRSA	IVa	SAMN49528718	SRR34096548
BGB564	invasive disease	5	CC5	2017	MRSA	IIa	SAMN49528719	SRR34096547
BGB565	invasive disease	5	CC5	2017	MRSA	IIa	SAMN49528720	SRR34096546
BGB566	invasive disease	5	CC5	2017	MSSA	-	SAMN49528721	SRR34096437
BGB567	invasive disease	5	CC5	2017	MRSA	IIa	SAMN49528722	SRR34096436
BGB568	invasive disease	8	CC8	2017	MRSA	IVa	SAMN49528723	SRR34096435
BGB569	invasive disease	6385	CC97	2017	MSSA	-	SAMN49528724	SRR34096434
BGB56B	invasive disease	8	CC8	2017	MRSA	IVa	SAMN49528725	SRR34097022
BGB56C	invasive disease	4236	CC5	2017	MSSA	-	SAMN49528726	SRR34097020

BGB56D	invasive disease	20	NoComplex	2017	MSSA	-	SAMN49528727	SRR34097019
BGB56F	invasive disease	30	CC30	2017	MSSA	-	SAMN49528728	SRR34097018
BGB56H	invasive disease	8	CC8	2017	MRSA	IVa	SAMN49528729	SRR34097017
BGB56J	invasive disease	8	CC8	2017	MRSA	IVa	SAMN49528730	SRR34097016
BGB56K	invasive disease	15	CC15	2017	MSSA	-	SAMN49528731	SRR34097015
BGB56L	invasive disease	5	CC5	2017	MSSA	-	SAMN49528732	SRR34097014
BGB56N	invasive disease	25	NoComplex	2017	MSSA	-	SAMN49528733	SRR34097013
BGB56P	invasive disease	8	CC8	2017	MRSA	IVa	SAMN49528734	SRR34097012
BGB56R	invasive disease	5	CC5	2017	MRSA	IIa	SAMN49528735	SRR34097011
BGB56S	invasive disease	15	CC15	2017	MSSA	-	SAMN49528736	SRR34097009
BGB56T	invasive disease	6386	NoComplex	2017	MSSA	-	SAMN49528737	SRR34097008
BGB56V	invasive disease	8	CC8	2017	MRSA	IVa	SAMN49528738	SRR34097007
BGB56X	invasive disease	72	CC8	2017	MSSA	-	SAMN49528739	SRR34097006
BGB56Y	invasive disease	8	CC8	2017	MRSA	IVa	SAMN49528740	SRR34097005
BGB56Z	invasive disease	5	CC5	2017	MSSA	-	SAMN49528741	SRR34097004
BGB570	invasive disease	8	CC8	2017	MRSA	IVa	SAMN49528742	SRR34097003
BGB572	invasive disease	188	CC1	2017	MSSA	-	SAMN49528743	SRR34097002
BGB573	invasive disease	9	CC1	2017	MSSA	-	SAMN49528744	SRR34097001
BGB574	invasive disease	8	CC8	2017	MRSA	IVa	SAMN49528745	SRR34097000
BGB575	invasive disease	398	NoComplex	2017	MSSA	-	SAMN49528746	SRR34096998
BGB576	invasive disease	5	CC5	2017	MSSA	-	SAMN49528747	SRR34096997
BGB577	invasive disease	8	CC8	2017	MRSA	IVa	SAMN49528748	SRR34096996
BGB578	invasive disease	188	CC1	2017	MSSA	-	SAMN49528749	SRR34096995
BGB57B	invasive disease	188	CC1	2017	MSSA	-	SAMN49528750	SRR34096994
BGB57C	invasive disease	8	CC8	2017	MRSA	IVa	SAMN49528751	SRR34096993
BGB57D	invasive disease	30	CC30	2017	MSSA	-	SAMN49528752	SRR34096992
BGB57F	invasive disease	8	CC8	2017	MRSA	IVa	SAMN49528753	SRR34096991
BGB57H	invasive disease	5	CC5	2017	MSSA	-	SAMN49528754	SRR34096882
BGB57J	invasive disease	8	CC8	2017	MRSA	IVa	SAMN49528755	SRR34096881
BGB57L	invasive disease	5	CC5	2017	MSSA	-	SAMN49528756	SRR34096879
BGB57M	invasive disease	87	NoComplex	2017	MRSA	Ivd	SAMN49528757	SRR34096878
BGB57N	invasive disease	5	CC5	2017	MSSA	-	SAMN49528758	SRR34096877
BGB57T	invasive disease	72	CC8	2017	MSSA	-	SAMN49528759	SRR34096876
BGB57W	invasive disease	5	CC5	2017	MRSA	IIa	SAMN49528760	SRR34096875
BGB57Y	invasive disease	1	CC1	2017	MSSA	-	SAMN49528761	SRR34096874
BGB57Z	invasive disease	188	CC1	2017	MSSA	-	SAMN49528762	SRR34096873
BGB580	invasive disease	4197	CC5	2017	MSSA	-	SAMN49528763	SRR34096872
BGB581	invasive disease	8	CC8	2017	MRSA	IVa	SAMN49528764	SRR34096871
BGB583	invasive disease	72	CC8	2017	MSSA	-	SAMN49528765	SRR34096870

BGB584	invasive disease	8	CC8	2017	MRSA	IVa	SAMN49528766	SRR34096868
BGB585	invasive disease	8	CC8	2017	MRSA	IVa	SAMN49528767	SRR34096867
BGB586	invasive disease	188	CC1	2017	MSSA	-	SAMN49528768	SRR34096866
BGB587	invasive disease	8	CC8	2017	MSSA	-	SAMN49528769	SRR34096865
BGB588	invasive disease	72	CC8	2017	MSSA	-	SAMN49528770	SRR34096864
BGB589	invasive disease	8	CC8	2017	MRSA	IVa	SAMN49528771	SRR34096863
BGB58B	invasive disease	188	CC1	2017	MSSA	-	SAMN49528772	SRR34096862
BGB58D	invasive disease	5	CC5	2017	MSSA	-	SAMN49528773	SRR34096861
BGB58F	invasive disease	188	CC1	2017	MSSA	-	SAMN49528774	SRR34096860
BGB58G	invasive disease	45	CC45	2017	MSSA	-	SAMN49528775	SRR34096859
BGB58K	invasive disease	8	CC8	2017	MRSA	IVa	SAMN49528776	SRR34096857
BGB58L	invasive disease	8	CC8	2017	MRSA	IVa	SAMN49528777	SRR34096856
BGB58M	invasive disease	1159	CC8	2017	MSSA	-	SAMN49528778	SRR34096855
BGB58N	invasive disease	8	CC8	2017	MRSA	IVa	SAMN49528779	SRR34096854
BGB58P	invasive disease	188	CC1	2017	MSSA	-	SAMN49528780	SRR34096853
BGB58R	invasive disease	8	CC8	2017	MSSA	-	SAMN49528781	SRR34096852
BGB58S	invasive disease	188	CC1	2017	MSSA	-	SAMN49528782	SRR34096851
BGB58T	invasive disease	72	CC8	2017	MSSA	-	SAMN49528783	SRR34096850
BGB58V	invasive disease	97	CC97	2017	MSSA	-	SAMN49528784	SRR34096849
BGB58W	invasive disease	8	CC8	2017	MSSA	-	SAMN49528785	SRR34096848
BGB58X	invasive disease	8	CC8	2017	MRSA	IVa	SAMN49528786	SRR34096545
BGB58Y	invasive disease	8	CC8	2017	MRSA	IVa	SAMN49528787	SRR34096544
BGB58Z	invasive disease	8	CC8	2017	MRSA	IVa	SAMN49528788	SRR34096543
BGB590	invasive disease	1187	CC5	2017	MRSA	IIa	SAMN49528789	SRR34096542
BGB591	invasive disease	8	CC8	2017	MRSA	IVa	SAMN49528790	SRR34096541
BGB592	invasive disease	5	CC5	2017	MSSA	-	SAMN49528791	SRR34096540
BGB593	invasive disease	8	CC8	2017	MRSA	IVa	SAMN49528792	SRR34096539
BGB594	invasive disease	8	CC8	2017	MSSA	-	SAMN49528793	SRR34096538
BGB595	invasive disease	5	CC5	2017	MSSA	-	SAMN49528794	SRR34096537
BGB597	invasive disease	8	CC8	2017	MRSA	IVa	SAMN49528795	SRR34096536
BGB598	invasive disease	72	CC8	2017	MSSA	-	SAMN49528796	SRR34096533
BGB599	invasive disease	1	CC1	2017	MRSA	IVa	SAMN49528797	SRR34096532
BGB59B	invasive disease	8	CC8	2017	MRSA	IVa	SAMN49528798	SRR34096531
BGB59C	invasive disease	8	CC8	2017	MRSA	IVa	SAMN49528799	SRR34096530
BGB59D	invasive disease	8	CC8	2017	MRSA	IVa	SAMN49528800	SRR34096529
BGB59F	invasive disease	6389	CC1	2017	MSSA	-	SAMN49528801	SRR34096528
BGB59G	invasive disease	5	CC5	2017	MSSA	-	SAMN49528802	SRR34096527
BGB59H	invasive disease	8	CC8	2017	MRSA	IVa	SAMN49528803	SRR34096526
BGB59J	invasive disease	1181	CC8	2017	MSSA	-	SAMN49528804	SRR34096525

BGB59K	invasive disease	5	CC5	2017	MSSA	-	SAMN49528805	SRR34096524
BGB59L	invasive disease	72	CC8	2017	MSSA	-	SAMN49528806	SRR34096522
BGB59M	invasive disease	1	CC1	2017	MRSA	IVa	SAMN49528807	SRR34096521
BGB59N	invasive disease	1	CC1	2017	MSSA	-	SAMN49528808	SRR34096520
BGB59P	invasive disease	5	CC5	2017	MSSA	-	SAMN49528809	SRR34096519
BGB59R	invasive disease	97	CC97	2017	MRSA	IVa	SAMN49528810	SRR34096518
BGB59S	invasive disease	97	CC97	2017	MSSA	-	SAMN49528811	SRR34096517
BGB59T	invasive disease	5	CC5	2017	MSSA	-	SAMN49528812	SRR34096516
BGB59V	invasive disease	188	CC1	2017	MSSA	-	SAMN49528813	SRR34096515
BGB59W	invasive disease	8	CC8	2017	MSSA	-	SAMN49528814	SRR34096514
BGB59X	invasive disease	188	CC1	2017	MSSA	-	SAMN49528815	SRR34096513
BGB59Y	invasive disease	45	CC45	2017	MSSA	-	SAMN49528816	SRR34096511
BGB59Z	invasive disease	5	CC5	2017	MSSA	-	SAMN49528817	SRR34096510
BGB5B1	invasive disease	72	CC8	2017	MSSA	-	SAMN49528818	SRR34096990
BGB5B2	invasive disease	1	CC1	2017	MSSA	-	SAMN49528819	SRR34096989
BGB5B3	invasive disease	105	CC5	2017	MRSA	IIa	SAMN49528820	SRR34096988
BGB5B4	invasive disease	188	CC1	2017	MSSA	-	SAMN49528821	SRR34096987
BGB5B5	invasive disease	8	CC8	2017	MRSA	IVa	SAMN49528822	SRR34096986
BGB5B6	invasive disease	8	CC8	2017	MRSA	IVa	SAMN49528823	SRR34096985
BGB5B8	invasive disease	188	CC1	2017	MSSA	-	SAMN49528824	SRR34096984
BGB5B9	invasive disease	12	NoComplex	2017	MSSA	-	SAMN49528825	SRR34096983
BGB5BC	invasive disease	188	CC1	2017	MSSA	-	SAMN49528826	SRR34096981
BGB5BD	invasive disease	1	CC1	2017	MRSA	IVa	SAMN49528827	SRR34096980
BGB5BF	invasive disease	97	CC97	2017	MSSA	-	SAMN49528828	SRR34096979
BGB5BG	invasive disease	45	CC45	2017	MSSA	-	SAMN49528829	SRR34096978
BGB5BJ	invasive disease	72	CC8	2017	MSSA	-	SAMN49528830	SRR34096977
BGB5BK	invasive disease	72	CC8	2017	MSSA	-	SAMN49528831	SRR34096976
BGB5BL	invasive disease	8	CC8	2017	MRSA	IVa	SAMN49528832	SRR34096975
BGB5BM	invasive disease	6372	CC8	2017	MRSA	IVa	SAMN49528833	SRR34096974
BGB5BP	invasive disease	188	CC1	2017	MSSA	-	SAMN49528834	SRR34096973
BGB5BR	invasive disease	188	CC1	2017	MSSA	-	SAMN49528835	SRR34096972
BGB5BS	invasive disease	8	CC8	2017	MRSA	IVa	SAMN49528836	SRR34096970
BGB5BT	invasive disease	1181	CC8	2017	MSSA	-	SAMN49528837	SRR34096969
BGB5BV	invasive disease	45	CC45	2017	MSSA	-	SAMN49528838	SRR34096968
BGB5BX	invasive disease	45	CC45	2017	MSSA	-	SAMN49528839	SRR34096967
BGB5BY	invasive disease	1	CC1	2017	MSSA	-	SAMN49528840	SRR34096966
BGB5BZ	invasive disease	8	CC8	2017	MSSA	-	SAMN49528841	SRR34096965
BGB5C0	invasive disease	8	CC8	2017	MRSA	IVa	SAMN49528842	SRR34096964
BGB5C1	invasive disease	188	CC1	2017	MSSA	-	SAMN49528843	SRR34096963

BGB5C3	invasive disease	6392	CC5	2017	MSSA	-	SAMN49528844	SRR34096962
BGB5C4	invasive disease	188	CC1	2017	MSSA	-	SAMN49528845	SRR34096961
BGB5C5	invasive disease	5	CC5	2017	MSSA	-	SAMN49528846	SRR34096959
BGB5C6	invasive disease	87	NoComplex	2017	MSSA	-	SAMN49528847	SRR34096958
BGB5C7	invasive disease	6176	NoComplex	2017	MSSA	-	SAMN49528848	SRR34096957
BGB5C9	invasive disease	97	CC97	2017	MSSA	-	SAMN49528849	SRR34096956
BGB5CB	invasive disease	8	CC8	2017	MRSA	IVa	SAMN49528850	SRR34096955
BGB5CC	invasive disease	97	CC97	2017	MSSA	-	SAMN49528851	SRR34096846
BGB5CF	invasive disease	188	CC1	2017	MSSA	-	SAMN49528852	SRR34096845
BGB5CG	invasive disease	5	CC5	2017	MSSA	-	SAMN49528853	SRR34096844
BGB5CJ	invasive disease	5	CC5	2017	MRSA	IIa	SAMN49528854	SRR34096843
BGB5CK	invasive disease	72	CC8	2017	MSSA	-	SAMN49528855	SRR34096842
BGB5CL	invasive disease	188	CC1	2017	MSSA	-	SAMN49528856	SRR34096840
BGB5CM	invasive disease	45	CC45	2017	MSSA	-	SAMN49528857	SRR34096839
BGB5CN	invasive disease	72	CC8	2017	MSSA	-	SAMN49528858	SRR34096838
BGB5CR	invasive disease	188	CC1	2017	MSSA	-	SAMN49528859	SRR34096837
BGB5CS	invasive disease	8	CC8	2017	MSSA	-	SAMN49528860	SRR34096836
BGB5CT	invasive disease	188	CC1	2017	MSSA	-	SAMN49528861	SRR34096835
BGB5CV	invasive disease	12	NoComplex	2017	MSSA	-	SAMN49528862	SRR34096834
BGB5CW	invasive disease	8	CC8	2017	MRSA	IVa	SAMN49528863	SRR34096833
BGB5CX	invasive disease	5	CC5	2017	MSSA	-	SAMN49528864	SRR34096832
BGB5CY	invasive disease	1	CC1	2017	MRSA	IVa	SAMN49528865	SRR34096831
BGPL39	invasive disease	97	CC97	2016	MRSA	IVa	SAMN49528866	SRR34096829
BKNSK0	invasive disease	72	CC8	2018	MSSA	-	SAMN49528867	SRR34096828
BKNSK1	invasive disease	30	CC30	2018	MSSA	-	SAMN49528868	SRR34096827
BKNSK2	invasive disease	8	CC8	2018	MSSA	-	SAMN49528869	SRR34096826
BKNSK3	invasive disease	8	CC8	2018	MRSA	IVa	SAMN49528870	SRR34096825
BKNSK4	invasive disease	188	CC1	2018	MSSA	-	SAMN49528871	SRR34096824
BKNSK5	invasive disease	8	CC8	2018	MRSA	IVa	SAMN49528872	SRR34096823
BKNSK7	invasive disease	5	CC5	2018	MSSA	-	SAMN49528873	SRR34096822
BKNSK8	invasive disease	6393	CC1	2018	MSSA	-	SAMN49528874	SRR34096821
BKNSK9	invasive disease	8	CC8	2018	MSSA	-	SAMN49528875	SRR34096820
BKNSKB	invasive disease	188	CC1	2018	MSSA	-	SAMN49528876	SRR34096818
BKNSKC	invasive disease	6358	CC8	2018	MSSA	-	SAMN49528877	SRR34096817
BKNSKD	invasive disease	51	NoComplex	2018	MSSA	-	SAMN49528878	SRR34096816
BKNSKF	invasive disease	30	CC30	2018	MSSA	-	SAMN49528879	SRR34096815
BKNSKH	invasive disease	8	CC8	2018	MRSA	IVa	SAMN49528880	SRR34096814
BKNSKJ	invasive disease	30	CC30	2018	MSSA	-	SAMN49528881	SRR34096813
BKNSKK	invasive disease	6394	CC8	2018	MSSA	-	SAMN49528882	SRR34096812

BKNSKL	invasive disease	8	CC8	2018	MRSA	IVa	SAMN49528883	SRR34096811
BKNSKM	invasive disease	97	CC97	2018	MSSA	-	SAMN49528884	SRR34096666
BKNSKN	invasive disease	548	CC8	2018	MSSA	-	SAMN49528885	SRR34096665
BKNSKP	invasive disease	97	CC97	2018	MSSA	-	SAMN49528886	SRR34096663
BKNSKR	invasive disease	188	CC1	2018	MSSA	-	SAMN49528887	SRR34096662
BKNSKS	invasive disease	5	CC5	2018	MSSA	-	SAMN49528888	SRR34096661
BKNSKT	invasive disease	8	CC8	2018	MRSA	IVa	SAMN49528889	SRR34096660
BKNSKV	invasive disease	398	NoComplex	2018	MRSA	Vc	SAMN49528890	SRR34096659
BKNSKW	invasive disease	97	CC97	2018	MSSA	-	SAMN49528891	SRR34096658
BKNSKX	invasive disease	6395	CC5	2018	MSSA	-	SAMN49528892	SRR34096657
BKNSKY	invasive disease	25	NoComplex	2018	MSSA	-	SAMN49528893	SRR34096656
BKNSKZ	invasive disease	672	NoComplex	2018	MSSA	-	SAMN49528894	SRR34096655
BKNSL0	invasive disease	30	CC30	2018	MSSA	-	SAMN49528895	SRR34096654
BKNSL3	invasive disease	8	CC8	2018	MRSA	IVa	SAMN49528896	SRR34096651
BKNSL4	invasive disease	6396	CC8	2018	MSSA	-	SAMN49528897	SRR34096650
BKNSL5	invasive disease	8	CC8	2018	MRSA	IVa	SAMN49528898	SRR34096649
BKNSL6	invasive disease	8	CC8	2018	MRSA	IVa	SAMN49528899	SRR34096648
BKNSL7	invasive disease	8	CC8	2018	MRSA	IVa	SAMN49528900	SRR34096647
BKNSL8	invasive disease	5	CC5	2018	MRSA	IVg	SAMN49528901	SRR34096646
BKNSL9	invasive disease	97	CC97	2018	MSSA	-	SAMN49528902	SRR34096645
BKNSLB	invasive disease	188	CC1	2018	MSSA	-	SAMN49528903	SRR34096644
BKNSLD	invasive disease	8	CC8	2019	MRSA	IVa	SAMN49528904	SRR34096643
BKNSLF	invasive disease	8	CC8	2018	MRSA	IVa	SAMN49528905	SRR34096642
BKNSLG	invasive disease	-	NoComplex	2018	MSSA	-	SAMN49528906	SRR34096640
BKNSLH	invasive disease	6397	CC1	2018	MSSA	-	SAMN49528907	SRR34096639
BKNSLK	invasive disease	188	CC1	2019	MSSA	-	SAMN49528908	SRR34096638
BKNSLL	invasive disease	6	CC5	2019	MSSA	-	SAMN49528909	SRR34096637
BKNSLM	invasive disease	8	CC8	2019	MRSA	IVa	SAMN49528910	SRR34096636
BKNSLN	invasive disease	8	CC8	2019	MSSA	-	SAMN49528911	SRR34096635
BKNSLP	invasive disease	8	CC8	2019	MSSA	-	SAMN49528912	SRR34096634
BKNSLV	invasive disease	5	CC5	2019	MSSA	-	SAMN49528913	SRR34096633
BKNSLW	invasive disease	5	CC5	2019	MRSA	IIa	SAMN49528914	SRR34096632
BKNSLZ	invasive disease	8	CC8	2019	MRSA	IVa	SAMN49528915	SRR34096631
BKNSM0	invasive disease	59	NoComplex	2019	MSSA	-	SAMN49528916	SRR34096508
BKNSM1	invasive disease	7295	CC5	2019	MSSA	-	SAMN49528917	SRR34096507
BKNSM2	invasive disease	5	CC5	2019	MRSA	IIa	SAMN49528918	SRR34096506
BKNSM3	invasive disease	9	CC1	2019	MSSA	-	SAMN49528919	SRR34096505
BKNSM4	invasive disease	25	NoComplex	2019	MSSA	-	SAMN49528920	SRR34096504
BKNSM5	invasive disease	72	CC8	2019	MSSA	-	SAMN49528921	SRR34096503

BKNSM6	invasive disease	8	CC8	2019	MRSA	IVa	SAMN49528922	SRR34096502
BKNSM7	invasive disease	97	CC97	2019	MSSA	-	SAMN49528923	SRR34096501
BKNSM8	invasive disease	5	CC5	2019	MSSA	-	SAMN49528924	SRR34096500
BKNSM9	invasive disease	8	CC8	2019	MSSA	-	SAMN49528925	SRR34096499
BKNSMB	invasive disease	188	CC1	2019	MSSA	-	SAMN49528926	SRR34096497
BKNSMF	invasive disease	97	CC97	2019	MSSA	-	SAMN49528927	SRR34096496
BKNSMG	invasive disease	5	CC5	2019	MRSA	IIa	SAMN49528928	SRR34096495
BKNSMH	invasive disease	188	CC1	2019	MSSA	-	SAMN49528929	SRR34096494
BKNSMJ	invasive disease	30	CC30	2019	MSSA	-	SAMN49528930	SRR34096493
BKNSMK	invasive disease	8	CC8	2019	MRSA	IVa	SAMN49528931	SRR34096492
BKNSML	invasive disease	34	CC30	2019	MSSA	-	SAMN49528932	SRR34096491
BKNSMM	invasive disease	8	CC8	2019	MRSA	IVa	SAMN49528933	SRR34096490
BKNSMP	invasive disease	8	CC8	2019	MRSA	IVa	SAMN49528934	SRR34096489
BKNSMR	invasive disease	5	CC5	2019	MSSA	-	SAMN49528935	SRR34096488
BKNSMS	invasive disease	8	CC8	2019	MRSA	IVa	SAMN49528936	SRR34096486
BKNSMT	invasive disease	15	CC15	2019	MSSA	-	SAMN49528937	SRR34096485
BKNSMV	invasive disease	97	CC97	2019	MSSA	-	SAMN49528938	SRR34096484
BKNSMW	invasive disease	97	CC97	2019	MSSA	-	SAMN49528939	SRR34096483
BKNSMX	invasive disease	188	CC1	2019	MSSA	-	SAMN49528940	SRR34096482
BKNSMY	invasive disease	30	CC30	2019	MSSA	-	SAMN49528941	SRR34096481
BKNSMZ	invasive disease	188	CC1	2019	MSSA	-	SAMN49528942	SRR34096480
BKNSN0	invasive disease	72	CC8	2019	MSSA	-	SAMN49528943	SRR34096479
BKNSN1	invasive disease	8	CC8	2019	MRSA	IVa	SAMN49528944	SRR34096478
BKNSN3	invasive disease	72	CC8	2019	MSSA	-	SAMN49528945	SRR34096477
BKNSN4	invasive disease	8	CC8	2019	MRSA	IVa	SAMN49528946	SRR34096475
BKNSN5	invasive disease	188	CC1	2019	MSSA	-	SAMN49528947	SRR34096474
BKNSN6	invasive disease	188	CC1	2019	MSSA	-	SAMN49528948	SRR34096954
BKNSN8	invasive disease	50	NoComplex	2019	MSSA	-	SAMN49528949	SRR34096953
BKNSNB	invasive disease	8	CC8	2019	MRSA	IVa	SAMN49528950	SRR34096952
BKNSNC	invasive disease	188	CC1	2019	MSSA	-	SAMN49528951	SRR34096951
BKNSND	invasive disease	8	CC8	2019	MSSA	-	SAMN49528952	SRR34096950
BKNSNF	invasive disease	188	CC1	2019	MSSA	-	SAMN49528953	SRR34096949
BKNSNH	invasive disease	97	CC97	2019	MSSA	-	SAMN49528954	SRR34096948
BKNSNJ	invasive disease	5	CC5	2019	MSSA	-	SAMN49528955	SRR34096947
BKNSNK	invasive disease	188	CC1	2019	MSSA	-	SAMN49528956	SRR34096945
BKNSNL	invasive disease	8	CC8	2019	MRSA	IVa	SAMN49528957	SRR34096944
BKNSNM	invasive disease	8	CC8	2019	MSSA	-	SAMN49528958	SRR34096943
BKNSNN	invasive disease	6398	CC8	2019	MSSA	-	SAMN49528959	SRR34096942
BKNSNP	invasive disease	97	CC97	2019	MSSA	-	SAMN49528960	SRR34096941

BKNSNR	invasive disease	188	CC1	2019	MSSA	-	SAMN49528961	SRR34096940
BKNSNS	invasive disease	6370	CC8	2019	MRSA	IVa	SAMN49528962	SRR34096939
BKNSNT	invasive disease	6399	CC5	2019	MSSA	-	SAMN49528963	SRR34096938
BKNSNV	invasive disease	1186	CC5	2019	MSSA	-	SAMN49528964	SRR34096937
BKNSNW	invasive disease	8	CC8	2019	MRSA	IVa	SAMN49528965	SRR34096936
BKNSNX	invasive disease	8	CC8	2019	MRSA	IVa	SAMN49528966	SRR34096934
BKNSNY	invasive disease	97	CC97	2019	MRSA	IVa	SAMN49528967	SRR34096933
BKNSNZ	invasive disease	8	CC8	2019	MSSA	-	SAMN49528968	SRR34096932
BKNSP0	invasive disease	87	NoComplex	2019	MSSA	-	SAMN49528969	SRR34096931
BKNSP1	invasive disease	8	CC8	2019	MSSA	-	SAMN49528970	SRR34096930
BKNSP2	invasive disease	8	CC8	2019	MSSA	-	SAMN49528971	SRR34096929
BKNSP3	invasive disease	188	CC1	2019	MSSA	-	SAMN49528972	SRR34096928
BKNSP4	invasive disease	1	CC1	2019	MRSA	IVa	SAMN49528973	SRR34096927
BKNSP6	invasive disease	97	CC97	2019	MSSA	-	SAMN49528974	SRR34096926
BKNSP7	invasive disease	5	CC5	2019	MSSA	-	SAMN49528975	SRR34096925
BKNSP8	invasive disease	5	CC5	2019	MSSA	-	SAMN49528976	SRR34096923
BKNSPB	invasive disease	8	CC8	2019	MRSA	IVa	SAMN49528977	SRR34096922
BKNSPC	invasive disease	6177	CC5	2019	MRSA	IIa	SAMN49528978	SRR34096921
BKNSPD	invasive disease	8	CC8	2019	MRSA	IVa	SAMN49528979	SRR34096920
BKNSPF	invasive disease	8	CC8	2019	MSSA	-	SAMN49528980	SRR34096919
BKNSPG	invasive disease	97	CC97	2019	MSSA	-	SAMN49528981	SRR34096810
BKNSPH	invasive disease	5	CC5	2019	MSSA	-	SAMN49528982	SRR34096809
BKNSPJ	invasive disease	5	CC5	2019	MRSA	IIa	SAMN49528983	SRR34096808
BKNSPK	invasive disease	1970	CC45	2019	MSSA	-	SAMN49528984	SRR34096807
NP_067_G1	carriage	6	CC5	2017	MSSA	-	SAMN35040418	SRR24507727
NP_098_G1	carriage	6956	CC121	2017	MSSA	-	SAMN35040420	SRR24507725
NP_103_G1	carriage	188	CC1	2017	MSSA	-	SAMN15567962	SRR12343899 (S); SRR12343982 (L)
NP_107_G1	carriage	188	CC1	2017	MSSA	-	SAMN35040423	SRR24507722
NP_123_G1	carriage	1	CC1	2017	MRSA	Vb(5C2&5)	SAMN15567963	SRR12343898 (S); SRR12343981 (L)
NP_157_G1	carriage	4092	CC30	2017	MSSA	-	SAMN15567964	SRR12343897 (S); SRR12343980 (L)
NP_161_G1	carriage	15	CC15	2017	MSSA	-	SAMN15567965	SRR12343896 (S); SRR12343979 (L)
NP_164_G1	carriage	188	CC1	2017	MSSA	-	SAMN15567966	SRR12343895 (S); SRR12343978 (L)
NP_175_G1	carriage	7317	CC30	2017	MSSA	-	SAMN35040438	SRR24507959
NP_176_G1	carriage	30	CC30	2017	MSSA	-	SAMN15567967	SRR12343894 (S); SRR12343977 (L)
NP_181_G1	carriage	50	NoComplex	2017	MSSA	-	SAMN15567968	SRR12343893 (S); SRR12343976 (L)
NP_183_G1	carriage	7549	CC30	2017	MSSA	-	SAMN35040441	SRR24507956
NP_183_G2	carriage	7317	CC30	2017	MSSA	-	SAMN35040442	SRR24507955
NP_196_G1	carriage	7317	CC30	2017	MSSA	-	SAMN15567969	SRR12343931 (S); SRR12343857 (L)
NP_198_G1	carriage	5	CC5	2017	MSSA	-	SAMN15567970	SRR12343931 (S); SRR12343857 (L)

NP_202_G1	carriage	15	CC15	2017	MSSA	-	SAMN35040448	SRR24507948
NP_202_G2	carriage	188	CC1	2017	MSSA	-	SAMN35040449	SRR24507947
NP_202_G3	carriage	188	CC1	2017	MSSA	-	SAMN35040450	SRR24507946
NP_204_G1	carriage	188	CC1	2017	MSSA	-	SAMN35040451	SRR24507945
NP_204_G2	carriage	30	CC30	2017	MSSA	-	SAMN35040452	SRR24507944
NP_204_G3	carriage	5	CC5	2017	MSSA	-	SAMN35040453	SRR24507943
NP_209_G1	carriage	30	CC30	2017	MSSA	-	SAMN15567971	SRR12343889 (S); SRR12343972 (L)
NP_215_G1	carriage	7317	CC30	2017	MSSA	-	SAMN15567972	SRR12343931 (S); SRR12343857 (L)
NP_223_G1	carriage	7317	CC30	2017	MSSA	-	SAMN15567973	SRR12343931 (S); SRR12343857 (L)
NP_229_G1	carriage	7317	CC30	2017	MSSA	-	SAMN15567974	SRR12343931 (S); SRR12343857 (L)
NP_250_G1	carriage	25	NoComplex	2017	MSSA	-	SAMN35040473	SRR24507885
NP_250_G3	carriage	5	CC5	2017	MSSA	-	SAMN35040475	SRR24507883
NP_260_G1	carriage	30	CC30	2017	MSSA	-	SAMN35040476	SRR24507882
NP_269_G1	carriage	51	NoComplex	2017	MSSA	-	SAMN35040482	SRR24507875
NP_269_G2	carriage	5	CC5	2017	MSSA	-	SAMN35040483	SRR24507874
NP_285_G1	carriage	5	CC5	2017	MSSA	-	SAMN35040485	SRR24507872
NP_285_G2	carriage	5	CC5	2017	MSSA	-	SAMN35040486	SRR24507871
OP_003_G1	carriage	7317	CC30	2017	MSSA	-	SAMN35040488	SRR24507936
OP_004_G1	carriage	30	CC30	2017	MSSA	-	SAMN15567975	SRR12343885 (S); SRR12343968 (L)
OP_007_G1	carriage	188	CC1	2017	MSSA	-	SAMN15567976	SRR12343931 (S); SRR12343857 (L)
OP_017_G1	carriage	8	CC8	2017	MSSA	-	SAMN15567977	SRR12343883 (S); SRR12343966 (L)
OP_026_G1	carriage	72	CC8	2017	MSSA	-	SAMN15567978	SRR12343931 (S); SRR12343857 (L)
OP_030_G1	carriage	72	CC8	2017	MSSA	-	SAMN35040497	SRR24507927
OP_035_G1	carriage	188	CC1	2017	MSSA	-	SAMN15567979	SRR12343931 (S); SRR12343857 (L)
OP_042_G1	carriage	39	CC30	2017	MSSA	-	SAMN15567980	SRR12343931 (S); SRR12343857 (L)
OP_043_G1	carriage	45	CC45	2017	MSSA	-	SAMN15567981	SRR12343931 (S); SRR12343857 (L)
OP_052_G1	carriage	188	CC1	2017	MSSA	-	SAMN15567982	SRR12343931 (S); SRR12343857 (L)
OP_056_G1	carriage	72	CC8	2017	MSSA	-	SAMN15567983	SRR12343931 (S); SRR12343857 (L)
OP_063_G1	carriage	8	CC8	2017	MRSA	IVa(2B)	SAMN15567984	SRR12343931 (S); SRR12343857 (L)
OP_064_G1	carriage	5	CC5	2017	MSSA	-	SAMN35040516	SRR24507906
OP_066_G1	carriage	72	CC8	2017	MSSA	-	SAMN35040517	SRR24507837
OP_071_G1	carriage	188	CC1	2017	MSSA	-	SAMN15567985	SRR12343931 (S); SRR12343857 (L)
OP_082_G1	carriage	15	CC15	2017	MSSA	-	SAMN35040529	SRR24507822
OP_082_G3	carriage	188	CC1	2017	MSSA	-	SAMN35040531	SRR24507820
OP_085_G1	carriage	1	CC1	2017	MSSA	-	SAMN35040532	SRR24507819
OP_087_G1	carriage	5	CC5	2017	MSSA	-	SAMN15567986	SRR12343931 (S); SRR12343857 (L)
OP_098_G1	carriage	5	CC5	2017	MSSA	-	SAMN15567987	SRR12343931 (S); SRR12343857 (L)
OP_098_G4	carriage	7550	NoComplex	2017	MSSA	-	SAMN35040540	SRR24507810
OP_101_G1	carriage	8	CC8	2017	MSSA	-	SAMN15567988	SRR12343931 (S); SRR12343857 (L)

OP_107_G1	carriage	97	CC97	2017	MSSA	-	SAMN15567989	SRR12343931 (S); SRR12343857 (L)
OP_111_G1	carriage	20	NoComplex	2017	MSSA	-	SAMN35040548	SRR24507769
OP_129_G1	carriage	72	CC8	2017	MSSA	-	SAMN15567990	SRR12343931 (S); SRR12343857 (L)
OP_131_G1	carriage	5	CC5	2017	MSSA	-	SAMN15567991	SRR12343931 (S); SRR12343857 (L)
OP_132_G1	carriage	188	CC1	2017	MSSA	-	SAMN15567992	SRR12343931 (S); SRR12343857 (L)
OP_133_G1	carriage	8	CC8	2017	MRSA	IVa(2B)	SAMN15567993	SRR12343931 (S); SRR12343857 (L)
OP_138_G1	carriage	188	CC1	2017	MSSA	-	SAMN35040560	SRR24507756
OP_138_G2	carriage	188	CC1	2017	MSSA	-	SAMN35040561	SRR24507755
OP_143_G1	carriage	20	NoComplex	2017	MSSA	-	SAMN15567994	SRR12343931 (S); SRR12343857 (L)
OP_143_G2	carriage	34	CC30	2017	MSSA	-	SAMN35040563	SRR24507753
OP_146_G1	carriage	6177	CC5	2017	MRSA	II(2A)	SAMN15567995	SRR12343863 (S); SRR12343946 (L)
OP_147_G1	carriage	188	CC1	2017	MSSA	-	SAMN15567996	SRR12343931 (S); SRR12343857 (L)
OP_153_G1	carriage	8	CC8	2017	MSSA	-	SAMN15567997	SRR12343931 (S); SRR12343857 (L)
OP_154_G1	carriage	5	CC5	2017	MSSA	-	SAMN15567998	SRR12343931 (S); SRR12343857 (L)
OP_163_G1	carriage	25	NoComplex	2017	MSSA	-	SAMN15567999	SRR12343859 (S); SRR12343942(L)

(S) short-read data; (L) long-read data.
